# The TGFβ/Notch axis facilitates Müller cell-to-epithelial transition to ultimately form a chronic glial scar

**DOI:** 10.1186/s13024-021-00482-z

**Published:** 2021-09-30

**Authors:** Federica Maria Conedera, Ana Maria Quintela Pousa, Nadia Mercader, Markus Tschopp, Volker Enzmann

**Affiliations:** 1 Department of Ophthalmology, University Hospital of Bern, University of Bern, Bern, Switzerland; 2 Department of BioMedical Research, University of Bern, Bern, Switzerland; 3 Graduate School for Cellular and Biomedical Sciences, University of Bern, Bern, Switzerland; 4 Advanced Microscopy Program, Center for Systems Biology, Massachusetts General Hospital, Boston, MA USA; 5 Wellman Center for Photomedicine, Massachusetts General Hospital, Boston, MA USA; 6 Institute of Anatomy, University of Bern, Bern, Switzerland; 7 Department of Ophthalmology, Cantonal Hospital Aarau, Aarau, Switzerland

**Keywords:** Laser injury, Müller cells, Notch pathway, Retinal degeneration, Retinal regeneration, Smad3, TGFβ signaling, Vertebrates

## Abstract

**Background:**

Contrasting with zebrafish, retinal regeneration from Müller cells (MCs) is largely limited in mammals, where they undergo reactive gliosis that consist of a hypertrophic response and ultimately results in vision loss. Transforming growth factor β (TGFβ) is essential for wound healing, including both scar formation and regeneration. However, targeting TGFβ may affect other physiological mechanisms, owing its pleiotropic nature. The regulation of various cellular activities by TGFβ relies on its interaction with other pathways including Notch. Here, we explore the interplay of TGFβ with Notch and how this regulates MC response to injury in zebrafish and mice. Furthermore, we aimed to characterize potential similarities between murine and human MCs during chronic reactive gliosis.

**Methods:**

Focal damage to photoreceptors was induced with a 532 nm diode laser in TgBAC (gfap:gfap-GFP) zebrafish (ZF) and B6-Tg (Rlbp1-GFP) mice. Transcriptomics, immunofluorescence, and flow cytometry were employed for a comparative analysis of MC response to laser-induced injury between ZF and mouse. The laser-induced injury was paired with pharmacological treatments to inhibit either Notch (DAPT) or TGFβ (Pirfenidone) or TGFβ/Notch interplay (SIS3). To determine if the murine laser-induced injury model translates to the human system, we compared the ensuing MC response to human donors with early retinal degeneration.

**Results:**

Investigations into injury-induced changes in murine MCs revealed TGFβ/Notch interplay during reactive gliosis. We found that TGFβ1/2 and Notch1/2 interact via Smad3 to reprogram murine MCs towards an epithelial lineage and ultimately to form a glial scar. Similar to what we observed in mice, we confirmed the epithelial phenotype of human Müller cells during gliotic response.

**Conclusion:**

The study indicates a pivotal role for TGFβ/Notch interplay in tuning MC stemness during injury response and provides novel insights into the remodeling mechanism during retinal degenerative diseases.

**Graphical abstract:**

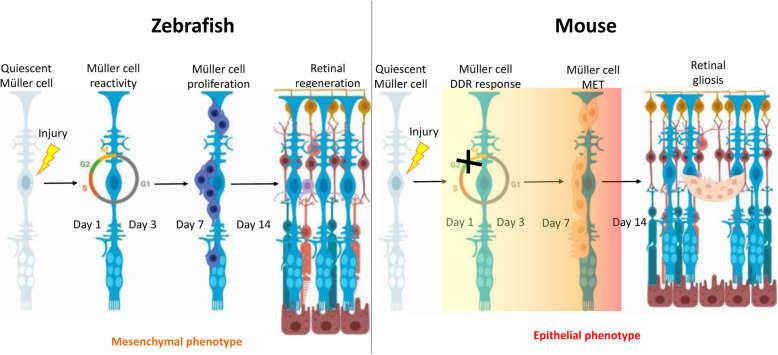

**Supplementary Information:**

The online version contains supplementary material available at 10.1186/s13024-021-00482-z.

## Background

Müller cells (MCs), major neuroglial cell type of the retina, behave as progenitor/stem cells upon injury in some vertebrates, but not in mammals [1]. Damage to zebrafish retina induces MC de-differentiation, proliferation, and generation of progenitors, which migrate to the damaged layer to restore it [2]. Contrariwise, mammalian MCs undergo reactive gliosis, a common feature of many retinal neurodegenerations. Reactive gliosis consists of the activation, proliferation and hypertrophic response of MCs following any injury/disease [3]. Initially, reactive gliosis protects retinal tissue from further damage. However, chronic reactive gliosis leads to the release of cytokines and mitogens implicated in various aspects of glial reactivity and scar formation, ultimately resulting in vision loss [4]. The complex molecular machinery that promotes retinal regeneration in teleost and glial scar formation in mammals is unknown [5]. Cross-species comparison between animal models with fully regenerative capacity and models with minimal/absent regenerative capacity can be beneficial to determine the molecular barrier of retinal regeneration in mammals. Recently, we showed that activation of either canonical or non-canonical TGFβ pathway is associated with dissimilar MC injury response in zebrafish and mice [6]. Each TGFβ isoform exerts a different effect on retinal tissue repair, which implies the pleiotropic nature of TGFβ action.

Accumulating evidence demonstrates that the regulation of various cellular activities by TGFβ relies on its interaction with other pathways [7]. Essential for tissue repair mechanisms in diverse organs and tissues (e.g., kidney, liver, and heart) is also the Notch pathway [8, 9]. Furthermore, many physiological as well as pathological processes that are regulated by Notch are also controlled by TGFβ, thus setting the stage for frequently occurring cross talk between the two pathways [10]. Here, we investigated TGFβ/Notch interplay during reactive gliosis and how they cooperatively regulate MC reactivity to injury in zebrafish and mice. Furthermore, we aimed to characterize and identify potential similarities between murine and human MCs during chronic reactive gliosis. Our findings illustrate the involvement of TGFβ/Notch interplay, via p-Smad3, during reactive gliosis, and how their combined action governs MC repair mechanism. Modulating these interactions may be a useful strategy to slow the progression of reactive gliosis in mammalian retina.

## Methods

### Animals

Adult TgBAC (gfap:gfap-GFP) zebrafish (> 6 month of age; AB strain; European Zebrafish Resource Center, Karlsruhe, Germany) have been used in this study [11]. They were kept under standard conditions in tank water and raised in a 14/10 h light/dark cycle. All new generations were monitored for GFP using a stereoscopic microscope [12]. Female and male B6-Tg (Rlbp1-GFP) mice (4–8 weeks old, originally provided by Prof. Dr. Christian Grimm) were kept in standard conditions with a 12-light/12-h dark cycle with food and water available ad libitum. Genotyping of Rlbp1-GFP mice was performed as previously described [6]. All animal experiments were approved by the local Animal Ethics Committee of the Canton Bern (Switzerland; BE34/19 and BE33/18) and conform to the Association for Research in Vision and Ophthalmology Statement for the Use of Animals in Ophthalmic and Vision Research.

### Human donor eyes

Retinal tissue from eight human donors (70–90-year-old) was used.

### Retinal laser focal injury

For both animal models, laser focal injury was induced as previously described [13]. Briefly, zebrafish were anesthetized with 0.16 mg/ml ethyl 3-aminobenzoate methanesulfonate salt (Tricaine; Sigma-Aldrich, Buchs, Switzerland) dissolved in tank water. A 532 nm diode laser (Visulas 532 s, Carl Zeiss Meditec AG, Oberkochen, Germany) was used to create lesions at the region of the posterior pole around the optic nerve. Four laser burns were applied to both eyes and the surrounding intact tissue was used as a negative control. For the RNAseq analysis, 20 laser burns were created. Each burn was produced with 70 mW of power for 100 ms and aimed to have a diameter of 50 μm. Mice were anesthetized by injecting subcutaneously 45 mg/kg ketamine (Ketalar 50 mg/ml; Orion Pharma AG, Zug, Zurich, Switzerland) and 0.75 mg/kg medetomidine hydrochloride (Domitor, 1 mg/ml; Orion Pharma AG). The same diode laser was used to create six lesions on the both eyes. For the RNAseq analysis, 50 laser burns were created in both eyes. Each burn was produced with 120 mW of power for 60 ms and aimed to be 100 μm in diameter.

### Spectral domain-optical coherence tomography (SD-OCT) and quantification

In vivo imaging of the murine retina was performed as previously described [6]. After anesthesia, pupils were dilated with a drop of tropicamide 0.5% phenylephrine 2.5% (ISPI, Bern, Switzerland), and methylcellulose (Methocel® 2%; OmniVision AG, Neuhausen, Switzerland) applied to each eye during imaging to keep them hydrated. Standard confocal laser scanning ophthalmoscope (Spectralis HRA + OCT; Heidelberg Engineering GmbH, Heidelberg, Germany) was used to image the murine retina [14]. SD-OCT was performed in both eyes using a 55° lens at a high resolution of 1008 × 596 pixels in grid mode. After imaging, 2.5 mg/kg atipamezole (Antisedan 5 mg/ml; Provet AG, Lyssach, Switzerland) was used to reverse the anesthesia. The area of each lesion was measured by using the Heidelberg Eye Explorer software (Heidelberg Engineering GmbH).

### Pharmacological cell-cycle arrest in zebrafish

Male and female zebrafish were randomly selected to be treated with palbociclib (PD0332991; Selleck Chemicals, Houston, TX, USA), a selective inhibitor of cyclin-dependent kinase (CDK) 4/6. The final concentration of 2 μM in tank water was based on a previous report [15]. Zebrafish were immersed at different timepoints (Day 4, 5 and 6) after injury induction and euthanized after 24 h (Day 5, 6 and 7, respectively). Injection paradigms are included in Figure S3. The negative control group was kept in tank water. Animals showing behavioral and/or morphological changes during treatment were excluded from the study.

### Pharmacological treatment in mice

Both male and female Rlbp1-GFP mice were randomly divided into three groups. The first group was treated with a γ-secretase inhibitor, (2S)-N-[(3,5-Difluorophenyl) acetyl]-L-alanyl-2-phenyl] glycine 1,1-dimethylethyl ester (DAPT; Tocris, Zug, Switzerland). DAPT powder was dissolved in dimethyl sulfoxide (DMSO; Sigma-Aldrich) and injected intraperitoneally (8 mg/kg body weight) either at 3 h before injury, at day 2, or at day 6 after injury. One day after injection, mice were euthanized (Day 1, 3 and 7, respectively) [16]. The second and the third group were treated with either 5-methyl-1-phenylpyridin-2-one (Pirfenidone; Selleckchem, Houston, TX, USA), which decreases the expression of TGFβ1/2/3 [17], or (*E*)-1-(6,7-dimethoxy-3,4-dihydro-1*H*-isoquinolin-2-yl)-3-(1-methyl-2-phenylpyrrolo [2,3-b]pyridin-3-yl)prop-2-en-1-one (SIS3; Selleckchem), a novel specific inhibitor of p-Smad3. Pirfenidone and SIS3 solutions were prepared according to previous studies [18], both drugs were dissolved in PBS containing 2% DMSO. Solutions were sonicated at 45 °C until transparent. Then 30% of polyethylene glycol (PEG)-300 (Med Lab Supply, Miami, FL, USA) was added to both. Furthermore, we added 2% Tween80 (Sigma-Aldrich) to the SIS3 solution only. Both mixtures were diluted with double distilled water (ddH2O) to 100 ml (5 mg/ml). Intraperitoneal injection dose was 50 mg/kg for pirferidone and 2.5 mg/kg for SIS3 at either 3 h before injury, at day 2 or at day 6. One day after injection, mice were euthanized (Day 1, 3 and 7, respectively). Injection paradigms are included in each figure (Fig. 5, S5, 6). Long-term treatment with SIS3 or control vehicle (Phosphate-buffered saline, PBS) was performed by intraperitoneal injection (2.5 mg/kg) at 3 h before injury, daily during the first three days after injury, and then every other day until day 14.

### Tissue processing and immunohistochemical studies

The eyes in both animal models were enucleated at different timepoints (Day 1, 3, 7 and 14) after injury and fixed with 4% paraformaldehyde (PFA) in PBS overnight. Human retina was fixed 1 h in 4% PFA in PBS. Paraffin sections (5 μm) were stained with Mayer’s hemalum and eosin (H&E; Roth, Karlsruhe, Germany) [13]; or used for immunofluorescence. Antigen retrieval was achieved by incubation in either Tris-EDTA (pH 9.0) or Citrate buffer (pH 6.0) with 0.05% Tween-20 for 20 min and then cooled at room temperature (~ 30 min). All sections were blocked for 1 h in Tris-buffered saline (TBS) + 5% goat normal serum + 1% bovine serum albumin (pH 7.6) and incubated with primary antibodies overnight at 4 °C. Primary antibodies used in this study were: mouse anti-glutamine synthetase (GS; 1:200; MAB302; Millipore, Billerica, MA, USA), rabbit anti-glutamine synthetase (GS; 1:200; ab210107; Abcam, Cambridge, UK), rabbit anti-glial fibrillary acidic protein (GFAP; 1∶200; OPA1–06100; ThermoFisher Scientific, Basel, Switzerland), rabbit anti-phospho extracellular signal-regulated kinase (Erk1/2; 1:100; 9101; Cell Signaling Technology, Danvers, MA, USA), mouse anti-proliferating cell nuclear antigen (PCNA; 1∶500; ab29; Abcam), rabbit anti-gamma histone H2A variant H2A.X (γH2A.X; 1∶200; ab228655; Abcam), rat anti-H2A.Z (1∶150; ab228655; Abcam), rabbit anti-phospho-histone 3 (pH 3; 1:150; 9713 T; Cell Signaling Technology), mouse anti-E Cadherin (1:200; ab76055; Abcam), rabbit anti-N Cadherin (1:500; ab18203; Abcam), rabbit anti-Notch homolog 1 (Notch1; 1:200; ab52627; Abcam), rabbit anti-Notch homolog 2 (Notch2; 1:100; D76A6; Cell Signaling Technology), rabbit anti-transforming growth factor beta 1 (Tgfβ1; 1:200 dilution; ab215715; Abcam), mouse anti-transforming growth factor beta 2 (Tgfβ2; 1:50 dilution; ab36495; Abcam), rabbit anti-transforming growth factor beta 3 (Tgfβ3; 1:100 dilution; ab15537; Abcam), rabbit anti-phosphorylated mothers against decapentaplegic homolog 3 (p-Smad3; 1:50 dilution; ab52903; Abcam), rabbit anti-orthodenticle homeobox 2 (Otx2; 1:200 dilution; ab183951; Abcam) and rabbit anti-paired box 6 (Pax6; 1:200 dilution; ab195045; Abcam). Secondary antibodies, goat anti-rabbit/anti-mouse Alexa 488 nm/594 nm (1∶500; ThermoFisher Scientific), were diluted in TBS with 1% BSA for 1 h at room temperature. Cell nuclei were counterstained using Vectashield with 4′, 6-diamidino-2-phenylindole (DAPI; Vector Labs, Burlingame, CA, USA).

### Flow cytometry analysis

At different timepoints after injury (Day 1, 3 and 7), retinas of gfap:gfap-GFP zebrafish and Rlbp1:GFP mice were used for flow cytometry analysis. Both retinas of each mouse were analyzed as one sample. Before antibody labeling, single cells suspensions were incubated with Hoechst 33342 cycling (ThermoFisher Scientific) in Hank’s Balanced Salt Solution (HBSS; ThermoFisher Scientific) with DNase I (200 U/ml; Sigma-Aldrich) to exclude dead cells. For antibody staining, the samples were washed, re-suspended in HBSS with 20% fetal bovine serum (FBS; ThermoFisher Scientific) and 200 U/ml DNase I. Reactive MCs were subsequently stained with fluorescent-labeled antibodies against GFAP (Alexa Fluor® 488 anti-GFAP antibody, 2E1.E9; Biolegend, San Diego, CA, USA), PCNA (PE anti-human/mouse/rat PCNA antibody, 307,908; Biolegend), Notch1 (Brilliant Violet 421™ anti-mouse Notch 1 antibody, 130,615; Biolegend) and with Notch2 (APC anti-mouse Notch 2 antibody, 130,713; Biolegend) at 4 °C in the dark for 40 min. Samples were washed again and re-suspended in 0.1% PFA (pH 7.4) at 4 °C in the dark for 10 min. Samples were washed twice, re-suspended in flow cytometry buffer, and analyzed. All washing steps involved addition of 1 ml HBSS with 0.01% DNase to each sample and centrifugation at 300 g at 4 °C for 3 min. Data were acquired with an LSR II Cytometer System and the BD FACSDiva software (BD Biosciences, Allschwil, Switzerland). The data were analyzed with the Flowjo Single Cell Analysis Software V10 (TreeStar, Ashland, OR, USA).

### Retinal dissociation, sorting, and RNA-Seq library production

Both retinas of three gfap:gfap-GFP zebrafish per timepoint (Day 1, 3, and 7) were dissected and dissociated in 0.05% trypsin (ThermoFisher Scientific) for 10 min and then suspended in DEPC-PBS with 10% FBS (ThermoFisher Scientific) and DNase I (200 U/ml; Sigma-Aldrich). Cell suspension was filtered and collected in Falcon® Round-Bottom Tubes with CellStrainer Cap (12 × 75 mm; Costar Corning, Cambridge, MA, USA). Hoechst 33342 Ready Flow™ Reagent (ThermoFisher Scientific) was added as DNA dye for cell-cycle analysis. Cells from gfap:gfap-GFP negative littermates were used to determine background fluorescence levels. 100 cells/μl were collected from gfap:gfap-GFP positive zebrafish using Moflo Astrias EQ Cell Sorter (Beckman Coulter, Brea, CA, USA) into 4 μl of Buffer TCL (Qiagen, Venlo, The Netherlands) with 1% 2-mercaptoethanol (#63,689; Sigma-Aldrich). Both retinas of three Rlbp1:GFP mice were dissected at different timepoints (Day 1, 3, and 7) and incubated with papain (Worthington Biochemical, Freehold, NJ, USA) for 15 min as previously described [19]. After dissociation, cell suspension in HBSS with 0.4% BSA (ThermoFisher Scientific) and DNase I (200 U/ml; Sigma-Aldrich) was filtered with a 35 μm cell strainer. Hoechst 33342 Ready Flow™ Reagent (ThermoFisher Scientific) was added as DNA dye for cell-cycle analysis. Cells from Rlbp1:GFP negative littermates were used to determine background fluorescence levels. 100 cells/μl were collected from Rlbp1:GFP positive mice using Moflo Astrias into 4 μl Buffer TCL (1,031,576; Qiagen) plus 1% 2-mercaptoethanol (Sigma-Aldrich). After cell sorting, all samples were processed using the published Smart-seq2 protocol to generate the cDNA libraries [20]. The libraries were sequenced in an Illumina HiSeq4000 (Illumina, San Diego, CA, USA) with a depth of around 20 Mio reads per sample. Sequencing data are available in the Gene Expression Omnibus database (NCBI tracking system #19961614).

### RNA-sequencing analysis

The raw reads were first cleaned by removing adapter sequences, trimming low quality ends, and filtering reads with low quality (phred quality < 20) using Trimmomatic (Version 0.36). The read alignment was done with STAR (v2.6.0c). As reference the Ensembl zebrafish genome build GRCz10 from 2017 to 06-07 (release 89) and respectively the Ensembl murine genome build GRCm38.p5 with the gene annotations downloaded on 2018-02-26 from Ensembl (release 91) were used. The STAR alignment options were “--outFilterType BySJout --outFilterMatchNmin 30 --outFilterMismatchNmax 10 --outFilterMismatchNoverLmax 0.05 --alignSJDBoverhangMin 1 --alignSJoverhangMin 8 --alignIntronMax 1000000 --alignMatesGapMax 1000000 --outFilterMultimapNmax 50”. Gene expression values were computed with the function featureCounts from the R package Rsubread (v1.26.0). The options for feature counts were: - min mapping quality 10 - min feature overlap 10 bp - count multi-mapping reads - count only primary alignments - count reads also if they overlap multiple genes. To detect differentially expressed genes, we applied a count based negative binomial model implemented in the software package DESeq2 (R version: 3.5.0, DESeq2 version: 1.20.0). The differential expression was assessed using an exact test adapted for over-dispersed data. Genes showing altered expression with an adjusted *p*-value < 0.05 (Benjamini and Hochberg method) were considered differentially expressed. Heatmaps were generated for selected subsets of genes in R v. 3.5.1 using the heatmap.2 function from package gplots v. 3.0.1. The data displayed the log2 fold-changes between two experimental groups. Rows are reordered based on a dendrogram from hierarchical clustering. Subsets of genes identified as interesting were explored using QIAGEN’s Ingenuity® Pathways Analysis suite (IPA®, QIAGEN, Redwood City, WA, USA;www.qiagen.com/ingenuity) for pathways, networks, and functional analyses.

### Image analysis and quantification

Immunofluorescence imaging was performed at 40x magnification with a scanning laser microscope (Zeiss LSM710; Carl Zeiss Microscopy, Jena, Germany). Sagittally oriented zebrafish and murine retinal sections at the level of the laser burns were used to quantify positive cells. The analyzed length of the retina was 50 μm in zebrafish or 100 μm in mouse, corresponding to the induced injury size. Arbitrary quantification of the central (fovea), mid-peripheral, and peripheral zones of each human retina was 606 μm in length (microscope’s visual field at 40x). The number of positive cells was normalized to the total number of MCs (cytoplasmatic GS^+^ or nuclear SOX9^+^) whereas the Ready Flow™ Reagent was normalized to the total number of DAPI positive cells in INL. Cells were manually determined. Ratios between positive cells on the total of MCs in the injured area were expressed as percentages. High-throughput and high-quality brightfield H&E-stained images of the human retina at 40x or 63x total magnification were acquired with a motorized Pannoramic 250 Flash II microscope (3DHISTECH Ltd., Budapest, Hungary). Randomized quantification of the central (fovea), mid-peripheral, and peripheral zones of each human retina was 950 μm in length (microscope’s visual field at 40x). Human samples were divided in two groups based on H&E and immunofluorescence data: control group (ctrl) and retina presenting drusen accumulation (drusen pos). Drusen were identified as accumulations of extracellular material that build up between Bruch’s membrane and the retinal pigment epithelium and manually counted. ImageJ software (v1.39; Wayne Rasband; NIH, Bethesda, MD, USA) was used to determine the length of the retina and analyze all images.

### Statistical analysis

Statistical analysis was performed using GraphPad Prism (version 7.0, GraphPad Software, La Jolla, USA). Intergroup comparisons were based on a non-parametric one−/two-way analysis of variance (ANOVA) and the Bonferroni multiple comparison post hoc test. For the pharmacologically treated animals, comparison between uninjured and treatment groups was performed with two-tailed t-test. Quantifications were performed on three laser burns performed in the left eye in four different animals for all timepoints (*n* = 12). All results are expressed as the mean ± standard deviation (SD). The level for statistical significance was set at a *p* value ≤0.05.

## Results

### Cross-species comparison of MC injury response

**Fig. 1:**
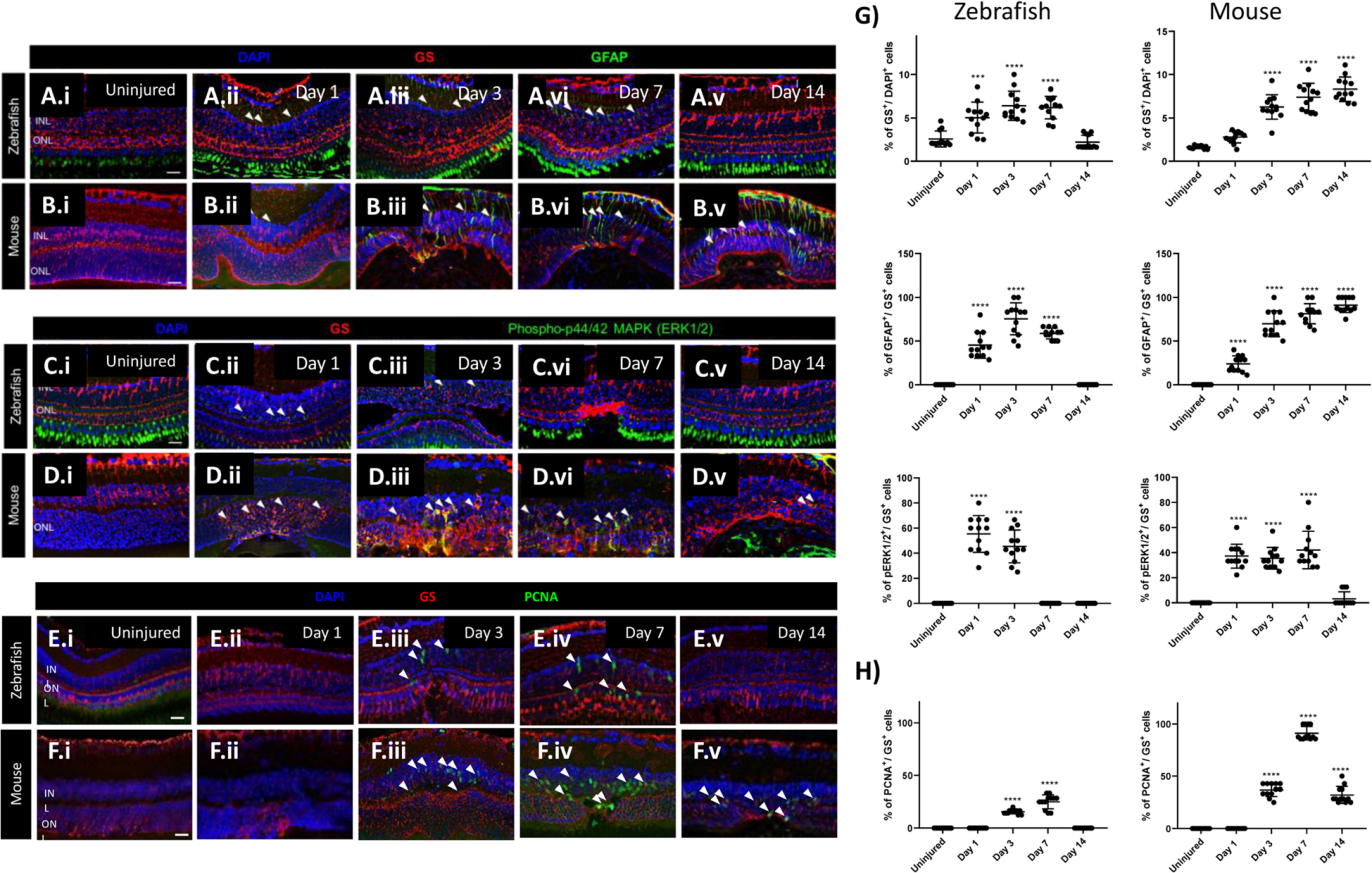
Cross-species comparison of MC gliosis in response to laser injury. (A-E) Analysis of MC gliotic response in zebrafish and mice at baseline (Uninjured) and different time point after injury (Day 1, 3, 7 and 14). Detection of GFAP in GS^+^MCs after laser induction in zebrafish (A.i-A.v) and mice (B.i-B.v). Shown are sections for GS (red) and GFAP (green). Detection of phospho-Erk1/2 in GS^+^MCs after injury and in uninjured zebrafish (C.i-C.v) and mice (D.i-D.v). Shown are sections for GS (red) and phospho-Erk1/2 (green). Histograms illustrating mean ± SD of GS^+^, GFAP^+^ and pERK1/2^+^cells normalized by the total of DAPI^+^ or GS^+^ cells in percentage (G). Significant differences (***p < 0.001, ****p < 0.0001) between uninjured and injured retinas were determined by post-hoc Bonferroni one-way ANOVA test (*n* = 12). (E-F; H) Evaluation of the proliferative potential of zebrafish and murine MCs during injury response at baseline (Uninjured) and different time point after injury (Day 1, 3, 7 and 14). Detection of PCNA in GS^+^MCs after injury in zebrafish (E.i-E.v) and mice (F.i-F.v). Shown are sections for GS (red) and PCNA (green). Histograms illustrating mean ± SD of PCNA^+^cells normalized by the total of GS^+^cells in percentage (H). Significant differences (****p < 0.0001) between uninjured and injured retinas were determined by post-hoc Bonferroni one-way ANOVA test (*n* = 12)

**Fig. 2:**
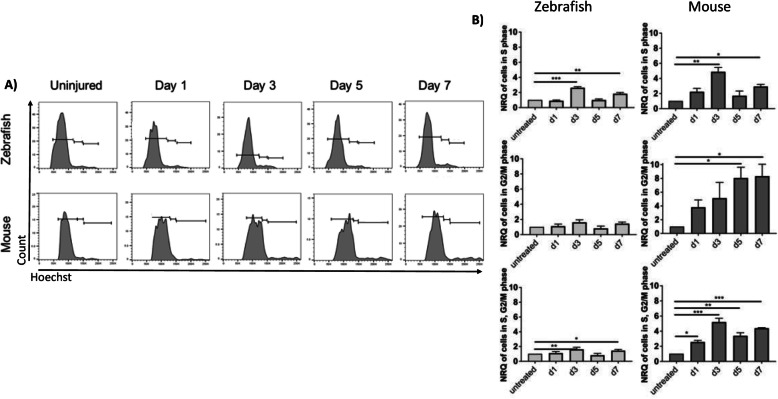
Analysis of cellular DNA content. (**A)** Flow cytometry of cellular DNA content (Hoechst) of zebrafish gfap:gfap-GFP^+^ and murine Rlbp1:GFP^+^ MCs. (**B)** Representative histograms illustrating mean ± SD of Hoechst^+^ cells in the different phases of the cell cycle normalized by the total of GFP^+^MCs in percentage at different time points (Day 1, 3, 5 and 7). Significant differences (*p < 0.05, **p < 0.01 and ***p < 0.001) between uninjured and injured at the different time points were determined by post-hoc Bonferroni one-way ANOVA test (*n* = 12). INL, inner nuclear layer; ONL, outer nuclear layer. Scale bar of the images equals 50 μm

To define MC reactivity in zebrafish and mice, we performed immunofluorescence for glutamine synthetase (GS), glial fibrillary acidic protein (GFAP) and phospho-p44/42 MAPK (phospho-Erk1/2) at days 1, 3, 7 and 14 after injury [21]. Uninjured retinas were compared to lasered ones at the different timepoints (Fig. 1A-E). GS was upregulated in zebrafish MCs within the damage area from day 1 (Fig. 1A.ii, E). The maximum GS expression was seen at day 3 (Fig. 1A.iii, E). When the retina was completely restored (Day 14), GS was comparable to baseline (Uninjured; Fig. 1A.v, E). MC reactivity was delayed in mice. GS expression in the injured area was upregulated starting from day 3 (Fig. 1B.iii, E) and increased further until day 14 (Fig. 1B.v, E). GFAP and phospho-Erk1/2 were upregulated from day 1 in GS^+^ MCs of both species (Fig. 1A.ii, B.ii, C.ii, D.ii, E). In zebrafish, GFAP at day 14 (Fig. 1A.v, E) and phospho-Erk1/2 from day 7 (Fig. 1C.iv, E) were no longer detectable. In mice, GFAP remains upregulated until day 14 (Fig. 1B.v), while phospho-Erk1/2 was downregulated at that time point (Fig. 1D.v, E). These data indicate a transient gliotic response in zebrafish and a persistent, respectively chronic, gliosis in mice after injury. To evaluate the proliferative potential of zebrafish and murine MCs during injury response, we analyzed proliferating cell nuclear antigen (PCNA) expression by immunohistochemistry (Day 1, 3, 7 and 14). Uninjured retinas were compared to lasered ones at different timepoints (Fig. 1F-H). From day 3, PCNA was detected in the GS^+^ MCs in both animal models (Fig. 1F.iii, G.iii, H). Whereas in zebrafish PCNA was no longer visible at day 14 (Fig. 1F.v, H), PCNA was still upregulated in murine MCs (Fig. 1G.v, H). PCNA is known as an S-phase marker used to detect proliferation [22]. Nevertheless, cells continue to express cell-cycle progression markers even upon DNA damage response (DDR) [23]. Thus, we examined the cellular DNA content in MCs by flow cytometry (Fig. 2A, B). In both animal models, we detected an increased number of MCs in S-phase at days 3 and 7 (Fig. 2A, B). However, quantification of cells in G2/M-phase showed that MCs accumulated in G2/M-phase exclusively in mice (Fig. 2A, B). These data suggest that murine MCs in response to injury are not able to properly segregate the duplicated genome leading to arrested re-entry into mitosis. Cells can be forced to exit the cell-cycle in response to DDR and become senescent [24]. Thus, we analyzed senescence-associated-DDR markers γH2A.X and H2A.Z in MCs (GS^+^) during injury response (Day 1, 3, 7 and 14). Retinas were compared at the different timepoints (Fig. S1 A-N). No DDR was detected in zebrafish MCs after injury (Fig. S1 A-C, G, H-J, N). However, in mice γH2A.X was upregulated at day 3 (Fig. S1 E.i-E.iv, G) and H2A.Z was observed at days 1 and 3 in MCs (Fig. S1 K.i-K.iv, L.i-L.iv, N). The cell-cycle arrest of murine MCs could be a consequence of DDR. The upregulation of H2A.Z may imply that abnormal MC behavior functions as a determinant of resistance to DNA damaging agents such as an erroneous cell-cycle re-entry.

### Phenotypic characterization of MC injury response

**Fig. 3:**
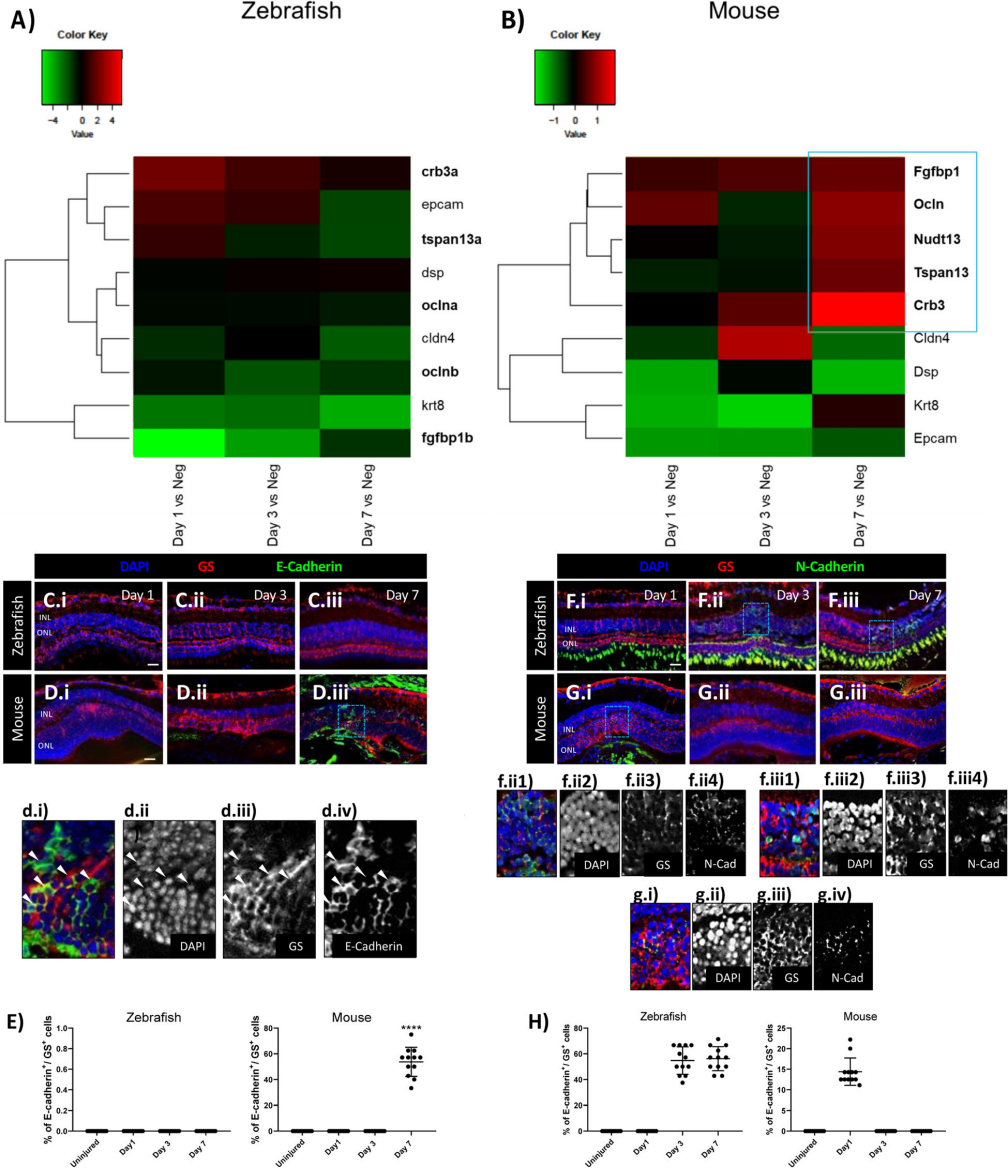
Difference of MC injury response in zebrafish and mice. (A, B) Heatmaps of differentially expressed genes (DEGs) associated with an epithelial phenotype in sorted cycling zebrafish and murine MCs. The blue box groups the most significant genes with the highest upregulation. (C-H) Analysis of MC phenotype in zebrafish and mice at the baseline (Uninjured) and at different time points after injury (Day 1, 3, and 7). Detection of E-cadherin in GS+ MCs after laser induction in zebrafish (C.i-C.iii) and mice (D.i-D.iii). Shown are retinal sections stained for GS (red) and E-cadherin (green). Zoomed-in view of murine GS+/E-cadherin+ cells of the area defined by a blue frame at Day 7 (d.i-d.iv). White arrowheads mark double-positive cells. (E) Histograms illustrating the mean ± SD of the number of E-cadherin+ cells normalized by the total number of GS+ cells expressed in percentage. Significant differences (****p < 0.0001) between uninjured and injured retinas were determined by using a post-hoc Bonferroni one-way ANOVA test (*n* = 12). Detection of N-Cadherin in GS+ MCs after laser induction in zebrafish (F.i-F.iii) and mice (G.i-G.iii). Shown are retinal sections stained for GS (red) and N-Cadherin (green). Zoomed-in view of zebrafish GS+/N-Cadherin+ cells of the area defined by a blue frame at Day 3 and 7 (f.ii1-f.ii4, f.iii1-f.iii4). Zoomed-in view of murine GS+/N-Cadherin+ cells of the area defined by a blue frame at Day 1 (g.i-g.iv). White arrowheads mark double-positive cells. (H) Histograms illustrating the mean ± SD of the number of N-Cadherin+ cells normalized by the total number of GS+ cells expressed in percentage. Significant differences (****p < 0.0001) between uninjured and injured retinas were determined by using a post-hoc Bonferroni one-way ANOVA test (*n* = 12)

Recent evidence indicates that mesenchymal-to-epithelial transition (MET) is crucial during early cell reprogramming and blocking MET can impair stem cell reprogramming [25]. Transcriptome analysis was used to investigate MET during early MC activation (Day 1), MC proliferation (Day 3), and MC regeneration/chronic reactive gliosis (Day 7) in both zebrafish and mice . We compared gene expression of zebrafish cycling gfap:gfap-GFP^+^ cells at days 1, 3 and 7 after injury with cycling gfap:gfap-GFP^+^ cells from uninjured retinas. In mice, we compared gene expression of cycling Rlbp1:GFP^+^ cells at days 1, 3 and 7 post injury with cycling MCs from uninjured retinas (Fig. 3A, B). In both animal models, we sorted for Müller cells in G2/M phase only. The term “cycling” was used to define the abnormal behavior of Müller cells, as they are forced to re-enter the cell-cycle but arrested in G2/M phase. Transcriptome analysis revealed an association of murine MCs with the acquisition of an epithelial phenotype at day 7 by the upregulation of epithelial-specific factors, as fibroblast growth factor binding protein 1 (Fgfbp1, 0.7 log2 ratio), occludin (Ocln; 1.02 log2 ratio), nudix hydrolase 13 (Nudt13; 0.95 log2 ratio), tetraspanin 13 (Tspan13; 0.8 log2 ratio), and crumbs cell polarity complex component 3 (Crb3; 1.88 log2 ratio). No epithelial markers were statistically modulated in zebrafish MCs (Fig. 3A). Thus, we analyzed whether murine MCs undergo epithelial-like changes during injury response. Pathway analysis was used to investigate major changes in gene expression during MC-to-epithelial transition (MC-ET; Fig. 3B). At day 7, we identified Wingless-Type MMTV Integration Site Family, Member 5B (Wnt5b; 1.84 log2 ratio), known to enable epithelialization [26], as one of the top listed genes to be upregulated in murine MCs (Fig. S2). This suggests that erroneous cell-cycle re-entry of MCs could be linked to epithelial remodeling during glial scar formation. To confirm murine MC-ET versus MC de-differentiation in zebrafish, we performed immunofluorescence for E- as well as N-cadherin during injury response (Day 1, 3, 7 and 14). Uninjured retinas were compared to injured retinas (Fig. 3C-H). We detected E-cadherin in murine GS^+^ MCs only at day 7, confirming MC-ET (Fig. 3D.iii, d.i-iv, E). In zebrafish, N-cadherin was upregulated in GS^+^ MCs from day 3 and stayed upregulated until day 7 (Fig. 3F.ii, f.ii1–4, Fiii, f.iii1–4, H). Interestingly, we observed N-cadherin also in murine MCs exclusively at day 1 (Fig. 3G.i, g.i-iv, H). These data showed the ability of zebrafish MCs to acquire a mesenchymal phenotype (N-cadherin) in response to injury versus MET (N- to E-cadherin shift) during reactive gliosis in the murine MCs.

### Notch pathway is linked with murine MC-ET

**Fig. 4:**
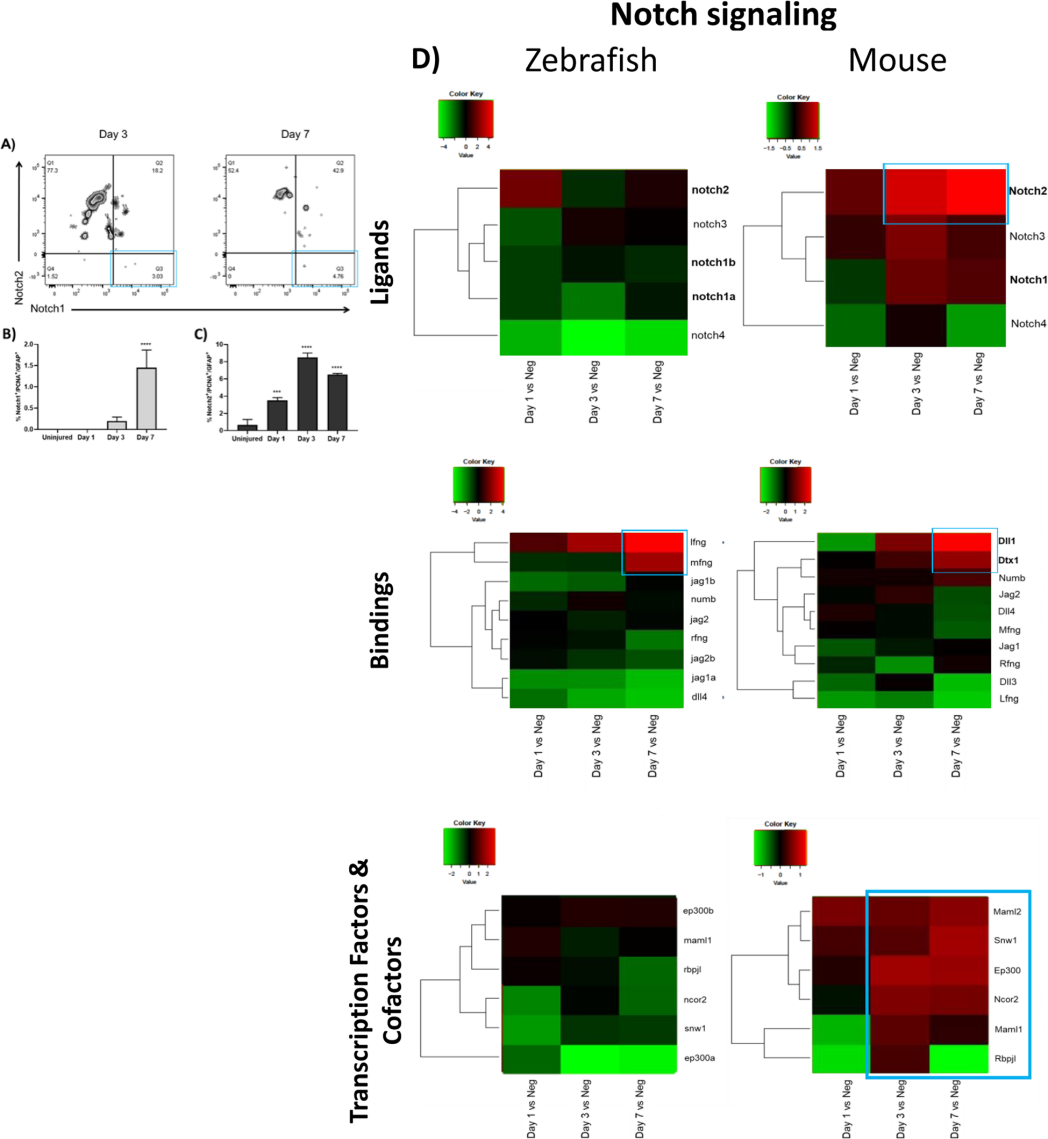
Investigation of Notch pathway during MET in murine MCs. Flow cytometry analysis of Notch isoforms in reactive MCs in mouse at the baseline (Uninjured) and at different time points after injury (Day 1, 3 and 7). (**A)** Representative figures of MCs gated for GFAP and PCNA and further for Notch1/2 at Day 3 and 7. (**B**-**C)** Histograms illustrating the mean ± SD of the number of GFAP+/PCNA+/Notch1+ cells (**B**) and GFAP+/PCNA+/Notch2+ cells (**C**) normalized by the total number of GFAP+/PCNA+ cells expressed in percentage. Significant differences (***p < 0.001 and ****p < 0.0001) between uninjured, Day 1, 3 and 7 were determined by using a post-hoc Bonferroni one-way ANOVA test (*n* = 12). (**D)** Heatmaps of receptors, ligands and transcription factors and cofactors of Notch signaling differentially expressed in sorted cycling zebrafish and murine MCs. Data are expressed as fold-changes compared to negative controls (cycling Müller from uninjured retinas). The blue boxes group the most significant genes with the highest upregulation

The mechanism that governs MET is regulated by numerous stimuli [27]. Notch is a key regulator of MET initiation. Therefore, we investigated Notch1/2 expression in reactive (GFAP^+^/PCNA^+^) MCs by flow cytometry in mice. Uninjured retinas were compared to different timepoints after injury (Day 1, 3 and 7; Fig. 4A-C). Notch1 was upregulated significantly at day 7 in a small percentage of reactive MCs (1.45% Notch1^+^/PCNA^+^/GFAP^+^ cells; Fig. 4A, B). Notch2 was detected from day 1 (3.5% Notch2^+^/PCNA^+^/GFAP^+^ cells) with the maximum expression at day 3 (8.5% Notch2^+^/PCNA^+^/GFAP^+^ cells; Fig. 4A, C). Notch pathway was studied by transcriptome analysis during early MC activation (Day 1), MC proliferation (Day 3), and MC regeneration/chronic reactive gliosis (Day 7) in both animal models. We compared the gene expression of zebrafish cycling gfap:gfap-GFP^+^ cells at days 1, 3 and 7 post injury with cycling gfap:gfap-GFP^+^ cells from uninjured retinas. In mice, we compared gene expression of cycling Rlbp1:GFP^+^ cells at days 1, 3 and 7 post injury with cycling MCs from uninjured retinas (Fig. 4D). None of Notch receptors were upregulated in zebrafish MCs after injury (Fig. 4D). Whereas, Notch2 was upregulated starting from day 3 (1.346 log2 ratio) with the maximum expression at day 7 (1.618 log2 ratio) in murine MCs (Fig. 4D). Additionally, we analyzed the expression of Notch ligands after injury in both animal models. In zebrafish MCs, we detected an upregulation of interferon gamma (ifng) and β-1,3-N-acetylglucosaminyltransferase manic fringe (mfng; Fig. 4D), genes known to orchestrate stem cell plasticity [28]. In murine MCs, we found an upregulation of delta-like canonical notch ligand 1 (Dll1) at day 3 (1.26 log2 ratio) and day 7 (2.62 log2 ratio), and an upregulation of deltex E3 ubiquitin ligase 1 (Dtx1) at day 7 only (1.513 log2 ratio; Fig. 4D). Interaction of Notch receptors with Dll1/Dtx1 may indicate the induction of γ-secretase-mediated cleavage, activating Notch cascade [29]. We further investigated Notch transcription factors and cofactors (Fig. 4D). None of them were upregulated in zebrafish MCs after injury (Fig. 4D). In murine MCs, nuclear receptor coactivator NCoA-62 (Snw1), required for MET [30], was upregulated at day 7 (0.8311 log2 ratio; Fig. 4D). These data suggest that Notch pathway may trigger murine MC-ET during injury response.

### Human MCs show an epithelial phenotype associated with TGFβ/notch under pathological condition

**Fig. 5:**
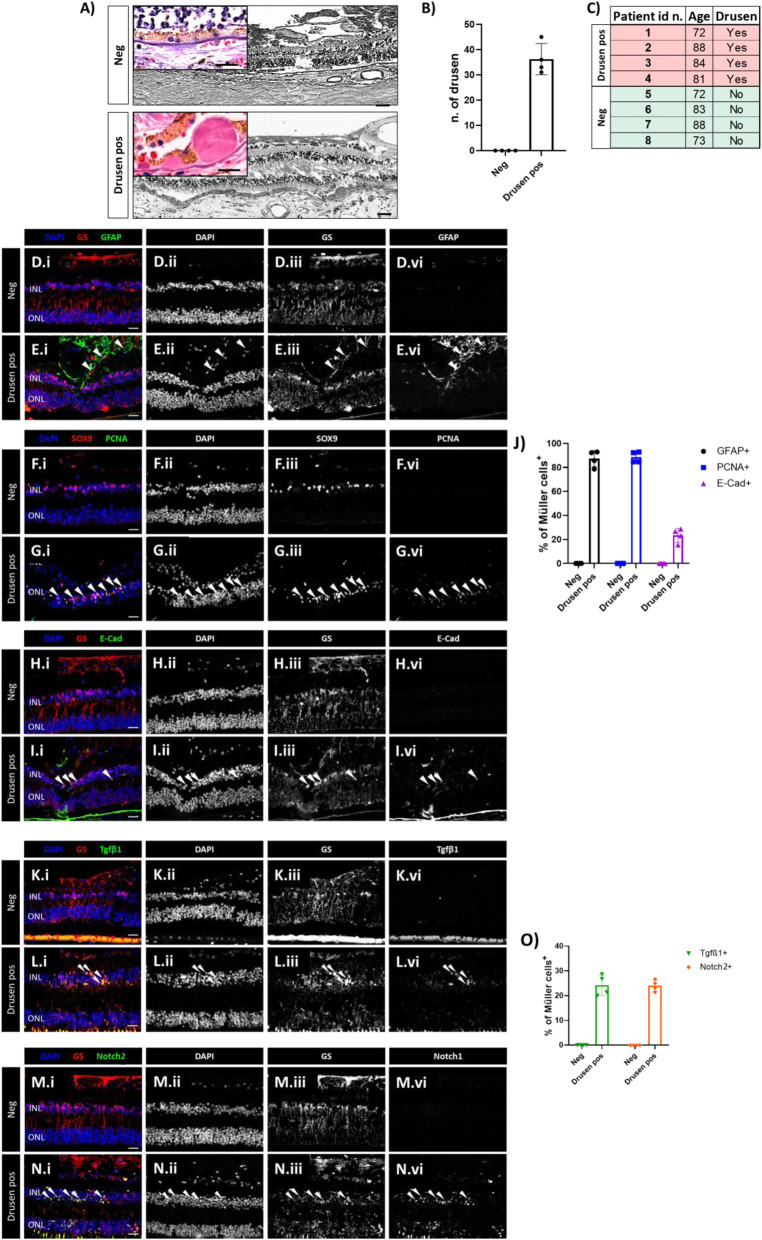
Human MCs show epithelial phenotype associated with TGFβ/Notch under pathological condition. (A) Human H&E stained sections of retinas that show healthy cuboidal RPE (Neg) and retinas presenting drusen beneath the pigment epithelium (Drusen pos). Zoom-in view showing a healthy cuboidal RPE layer (Neg, top left corner) and drusen or micro drusen underneath the RPE layer (Drusen pos, top left corner). (B) Quantification of drusen as either hyalinized rounded deposits (> 25 μm) or micro drusen occurring singly or in a row (< 25 μm) in between the RPE and the Bruch’s membrane. The analyzed length of the retina was 950 μm retina sections corresponding to the field of view. (C) Table summarizing the eight selected samples (total analyzed sections = 52) selected for H&E and immunofluorescence analysis. (D-J) Analysis of MC reactivity and phenotype of healthy retinas (Neg) and retinas presenting drusen (Drusen pos). Detection of GFAP (D.i-D.iv, E.i-E.iv), PCNA (F.i-F.iv, G.i-G.iv) and E-cadherin (H.i-H.iv, I.i-I.iv) in GS+ MCs. Shown are representative sections stained for GS and SOX9 (red), GFAP, PCNA and E-Cadherin (green). (J) Histogram illustrating the mean ± SD of the number of GFAP+, PCNA+ and E-cadherin+ cells normalized by the total number of GS+ cells expressed in percentage. Significant differences (****p < 0.0001) between “Neg” and “Drusen pos” retinas were determined by using a post-hoc Bonferroni two-way ANOVA test (*n* = 8). (K-O) Analysis of regulators of MET induction in healthy retinas (Neg) and retinas presenting drusen (Drusen pos). Detection of TGFβ1 (K.i-K.iv, L.i-L.iv) and Notch2 (M.i-M.iv, N.i-N.iv) in GS+ MCs. Shown are representative retinal sections stained for GS (red), TGFβ1, and Notch2 (green). (O) Histogram illustrating the mean ± SD of the number of TGFβ1+ and Notch2+ cells normalized by the total number of GS+ cells expressed in percentage. INL, inner nuclear layer; ONL, outer nuclear layer. Scale bar of all images equals 50 μm, while in the zoom-in view corresponding to 150 μm

Drusen are an early pathological feature of retinal degenerations (e.g., AMD [31];). We performed H&E staining on 52 human retinal samples and selected for comparison sections with healthy RPE layer and sections with drusen accumulation beneath the pigment epithelium (*n* = 8; Fig. 5A-C). The drusen were thereby detected as either hyalinized rounded deposits (> 25 μm) or micro drusen occurring singly or in a row (< 25 μm) between the pigment epithelium and the Bruch’s membrane. Samples are subdivided in two groups (4 each group): retinas that show healthy cuboidal RPE (Neg) and retinas presenting drusen beneath the pigment epithelium (Drusen pos; Fig. 5A-C). Both groups were tested for MC reactivity (GFAP and PCNA; Fig. 5D-G), epithelial marker (E-cadherin; Fig. 5H, I), and regulators of MC-ET induction (TGFβ1/Notch2; Fig. 5K-N) by immunofluorescence. MCs were identified by either cytosolic-GS or nuclear-SOX9 positivity. We detected significant upregulation of GFAP/PCNA in all retinas presenting drusen (Fig. 5E, G, J), confirming their gliotic state. Additionally, only retinas presenting drusen (Drusen pos) showed an upregulation of E-cadherin, TGFβ1, and Notch2 (Fig. 5I, J, L, N, O). This may suggest the acquisition of an epithelial phenotype by human reactive MCs, which is associated to TGFβ1/Notch2 expressions under pathological condition.

### Impact of erroneous cell-cycle arrest on retinal repair in zebrafish

To investigate the effects of an erroneous cell-cycle arrest on the regenerative potential of Müller cells (MCs), three different groups of zebrafish were treated (24 h) with palbociclib at days 4, 5 and 6, when the MCs are de-differentiating. The three different timepoints after treatment (Day 5, 6 and 7, respectively) were compared to each other (Fig. S3 A-D). We investigated cell-cycle progression in GS^+^ MCs by immunofluorescence for PCNA and phospho-Histone H3 (p-H3), known as a mitosis-specific marker. Additionally, we studied the DNA damage response (DDR) by HA2.X and HA2.Z staining. PCNA signal was upregulated at every timepoint investigated (Day 5, 6 and 7; Fig. S3 A.i-iv). Instead, p-H3 was not visible throughout the experiment confirming the efficiency of the pharmacological treatment (Day 5, 6 and 7; Fig. S3 B.i-iv). Therefore, zebrafish MCs irreversibly commit to the mitotic cell-cycle in an inactive state after treatment. Regarding DDR, we detected an upregulation of HA2.X, linking the DDR with an erroneous cell-cycle arrest induced in zebrafish MCs by palbociclib treatment at every timepoint investigated (Day 5, 6 and 7; Fig. S3 C.i-iv). However, HA2.Z was not observed, suggesting that cell-cycle arrest is not required to initiate repair mechanisms (Day 5, 6 and 7; Fig. S3 D.i-iv). These data suggest that induced cell-cycle arrest observed in zebrafish, as well as arrested re-entry into mitosis in mice, may be the trigger for DDR. However, cell-cycle arrest may not be linked with a proper assembly of a chromatin template, which is an efficient substrate for the DSB repair machinery. We studied the effect of palbociclib treatment on MC phenotype N-cadherin and connective tissue growth factor (CTGF), prominently elevated under fibrotic-like conditions such as gliosis [32] (Fig. S4 A.i-iv, B.i-iv). After induced cell-cycle arrest, zebrafish MCs within the damaged area adopted a pro-fibrotic phenotype throughout the experiment (Day 5, 6 and 7; Fig. S4 A.i-iv, B.i-iv). These data may link the induced cell-cycle arrest of zebrafish MCs to their expression of CTGF, known to be associated with pathological scarring in conditions such as fibrosis [33]. Furthermore, we investigated the expression of TGFβ isoforms (TGFβ1/2/3), Notch isoforms (Notch1/2), and pSmad3 after injury in palbociclib treated zebrafish at different timepoints (Day 4, 5 and 6; Fig. S4 C.i-iv, D.i-iv, E.i-iv, F.i-iv, G.i-iv, H.i-iv). Both Notch isoforms, were upregulated throughout the experiment (Day 5, 6 and 7; Fig. S4 C.i-iv, D.i-iv). Whereas, only TGFβ1 and 3 were upregulated after induced cell-cycle arrest in the injured area (Day 5, 6 and 7; Fig. S4 E.i-iv, F.i-iv, G.i-iv). The signal of pSmad3 was also detectable at every timepoint investigated (Day 5, 6 and 7; Fig. S4 H.i-iv). These data associate the expression of TGFβ isoforms, mainly TGFβ1, both Notch isoforms, and pSmad3 with cell-cycle arrest in palbociclib treated zebrafish as summarized in Fig. S4 I.

### Pharmacological TGFβ inhibition during injury response in murine MCs

MET can be induced or regulated by various growth factors involved in cell differentiation, as TGFβ, or act through receptor tyrosine kinases, as Notch [34]. Therefore, we suppressed the TGFβ pathway using pirfenidone at three different timepoints in mice: 3 h before injury (baseline), day 2 (MC-cycle arrest), and day 6 (MC-ET). Three different timepoints after injury (Day 1, 3 and 7) were compared to each other (Fig. S5 A-I). To evaluate the efficiency of TGFβ pathway inhibition by pirfenidone, we perform immunofluorescence for MC marker (GS^+^), TGFβ isoforms (TGFβ1/2/3) and, pSmad3 at different timepoints after injury (Day 1, 3 and 7; Fig. S5 A.i-iii, B i-iii, C i-iii, D i-iii). No signal of any TGFβ isoform as well as pSmad3 was visible throughout the experiment (Day 1, 3 and 7) in MCs, confirming the inhibition of TGFβ pathway during injury response following treatment (Day 5, 6 and 7; Fig. S5 A.i-iii, B i-iii, C i-iii, D i-iii). GS+ MC phenotype was analyzed by immunofluorescence for E- and N-Cadherin expression (Fig. S5 E.i-iii, F.i-iii). Both cadherins were not detectable in GS+ MCs of pirfenidone treated mice at any timepoint investigated (Day 1, 3 and 7; Fig. S5 E.i-iii, F.i-iii). These data suggest that TGFβ inhibition may maintain murine MCs in a quiescent state even after injury. Furthermore, we investigated how Notch pathway inhibition using pirfenidone affects the expression of Notch isoforms (Notch1/2) in GS+ MCs at different timepoints after injury (Day 1, 3 and 7) by immunofluorescence (Fig. S5 G.i-iii, H.i-iii). We did not detect a modulation of Notch isoforms within the injured area in MC following treatment (Fig. S5 G.i-iii, H.i-iii). Altogether, these data suggest the pivotal role of TGFβ during MCs during injury response and how TGFβ may regulate the expression of Notch pathway in murine MC in response to injury (Fig. S5 I).

### Pharmacological Notch inhibition during MC injury response

**Fig. 6:**
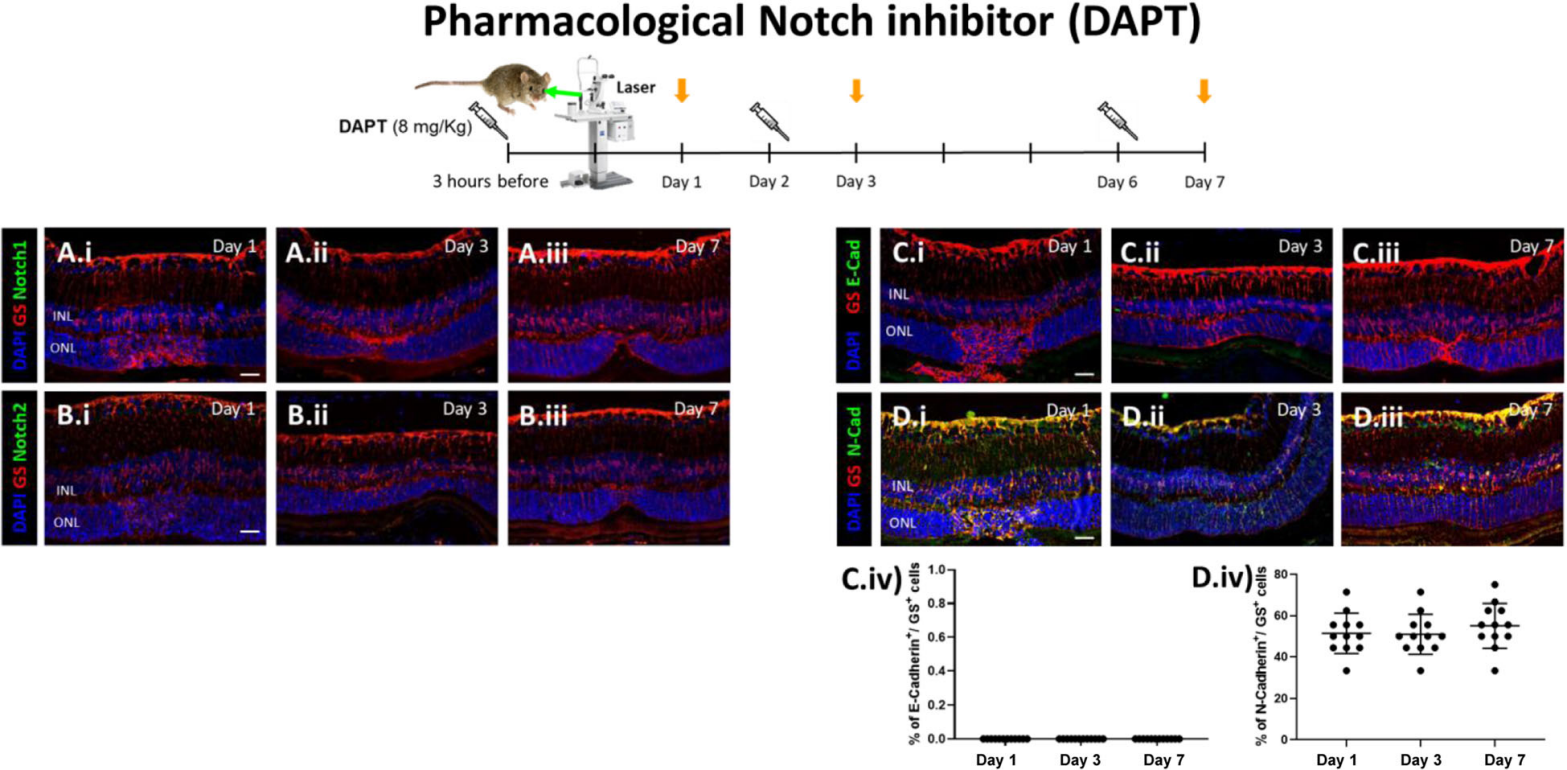
Pharmacological Notch inhibition (DAPT) increases N-cadherin expression. Mice were treated with DAPT (8 mg/kg) by intraperitoneal injection either at 3 h before injury, at day 2 or at day 6 (syringes) and euthanized 24 h after injection (orange arrows; Day 1, 3, 7, respectively). (A-B) Analysis of Notch isoforms during MC injury response in DAPT treated mice at different time points (Day 1, 3 and 7). Detection of Notch1 (A.i-A.iii) and Notch2 (B.i-B.iii) in GS^+^ MCs. Shown are representative sections stained for GS (red), Notch1/2 (green). (C-D) Analysis of MC phenotype during injury response in DAPT treated mice at different time points (Day 1, 3 and 7). Detection of E-cadherin (C.i-C.iii) and N-cadherin (D.i-D.iii) in GS+ MCs. Shown are representative sections stained for GS (red), E- and N-cadherin (green). (C.iv, D.iv) Histograms illustrating the mean ± SD of the number of Notch1^+^ and Notch2^+^ cells normalized by the total number of GS^+^ cells expressed in percentage (*n* = 12)

**Fig. 7:**
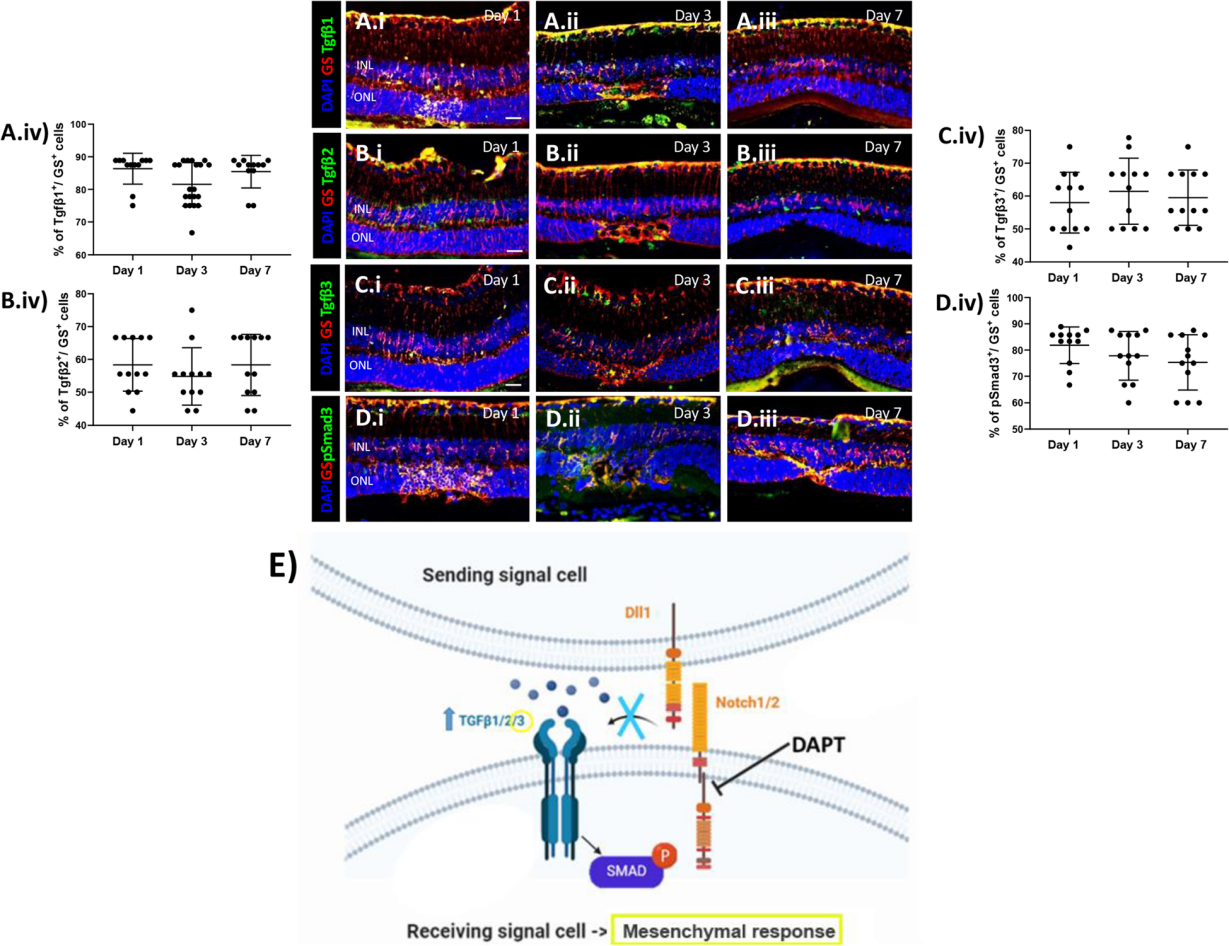
Pharmacological Notch inhibition (DAPT) increases TGFβ3 expression. (A-D) Analysis of TGFβ pathway during MC injury response in DAPT treated mice at different time points (Day 1, 3 and 7). Detection of TGFβ1 (A.i-A.iii), TGFβ2 (B.i-B.iii), TGFβ3 (C.i-C.iii) and p-Smad3 (D.i-D.iii) in GS^+^ MCs. Shown are representative sections stained for GS (red), TGFβ1/2/3 and p-Smad3 (green). (A.iv, B.iv, C.iv, D.iv) Histograms illustrating the mean ± SD of the number of TGFβ1, TGFβ2 and TGFβ3^+^ cells normalized by the total number of GS+ cells expressed in percentage (n = 12). INL, inner nuclear layer; ONL, outer nuclear layer. Scale bar of all images equals 50 μm. (E) Schematic summary of molecular outcomes of DAPT treatment in murine MCs

Notch pathway has been implicated in MET induction that is associated with fibrosis [35]. Thus, we inhibited Notch pathway using DAPT for 24 h in mice at 3 h before injury (baseline), day 2 (during MC-cycle arrest), and day 6 (MC-ET). The resulting timepoints of evaluation (Day 1, 3 and 7, respectively) were compared to each other (Figs. 6 and 7). To assess the efficiency of DAPT, we performed immunofluorescence for MC marker (GS^+^) and Notch1/2. Notch isoforms were not visible at any timepoint, confirming their inhibition during injury response (Day 1, 3 and 7; Fig. 6A.i-iii, B.i-iii). MC phenotype was analyzed by immunofluorescence for E- and N-cadherin. Thereby, E-cadherin was not visible in GS^+^ MCs at any timepoint after injury (Day 1, 3 and 7; Fig. 6C.i-iv). Instead, N-cadherin was upregulated at all timepoints (Day 1, 3 and 7; Fig. 6D.i-iv), suggesting that Notch inhibition may induce a mesenchymal response in murine MCs after injury. Furthermore, we investigated how DAPT affects the expression of TGFβ isoforms (TGFβ1/2/3) and p-Smad3 in GS^+^ MCs during injury response (Day 1, 3 and 7) by immunofluorescence analysis (Fig. 7A.i-iv, B.i-iv, C.i-iv, D.i-iv). All TGFβ isoforms along with p-Smad3 were upregulated in MCs. Altogether, these data may associate Notch inhibition with the expression of TGFβ isoforms, mainly TGFβ3, and p-Smad3, initiating a mesenchymal response in murine MCs in response to injury.

### Pharmacological Smad3 inhibition attenuates glial scar formation

**Fig. 8:**
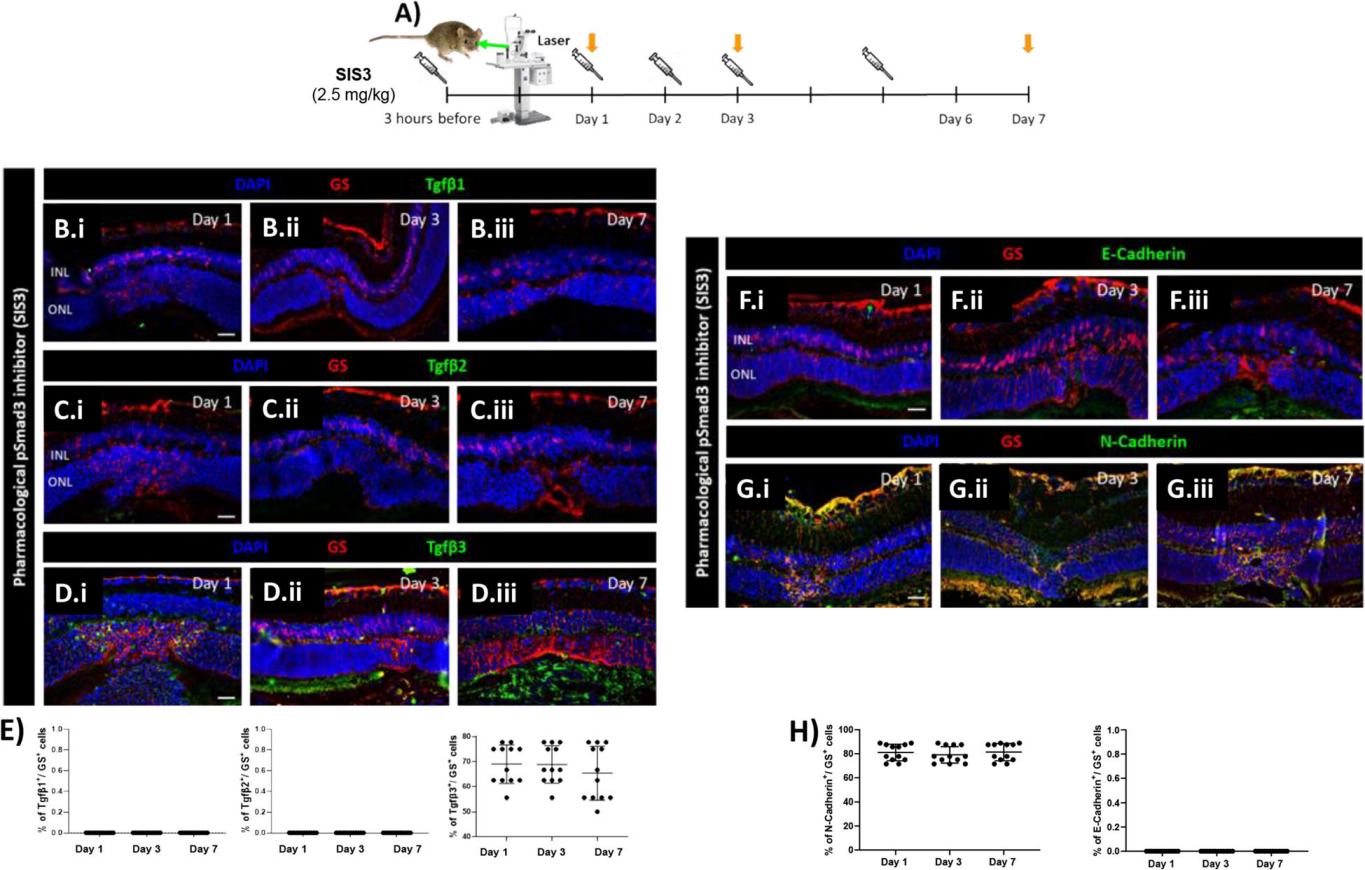
Pharmacological inhibition of p-Smad3 (SIS3) increase TGFβ3 and N-cadherin. (A) SIS3treated mice treated at 3 h before injury, at day 2 or at day 6 (syringes) and euthanized 24 h after injection (orange arrows; Day 1, 3, 7, respectively). (B-E) MC reactivity in SIS3 treated mice. Detection of TGFβ1 (B.i-B.iii), TGFβ2 (C.i-C.iii) and TGFβ3 (D.i-D.iii) in GS^+^MCs. Shown are representative sections for GS (red) and TGFβ1/2/3 (green). Histograms illustrating mean ± SD of TGFβ1, TGFβ2 and TGFβ3^+^cells normalized by total of GS^+^cells (E; n = 12). (F-H) MC phenotype in SIS3 treated mice. Detection of E-cadherin (F.i-F.iii) and N-cadherin (G.i-G.iii) in GS^+^MCs. Shown are representative sections stained for GS (red) as well as E- and N-cadherin (green). Histograms illustrating mean ± SD of E-cadherin^+^/N-cadherin^+^ cells normalized by the total of GS^+^ cells (H; n = 12)

**Fig. 9:**
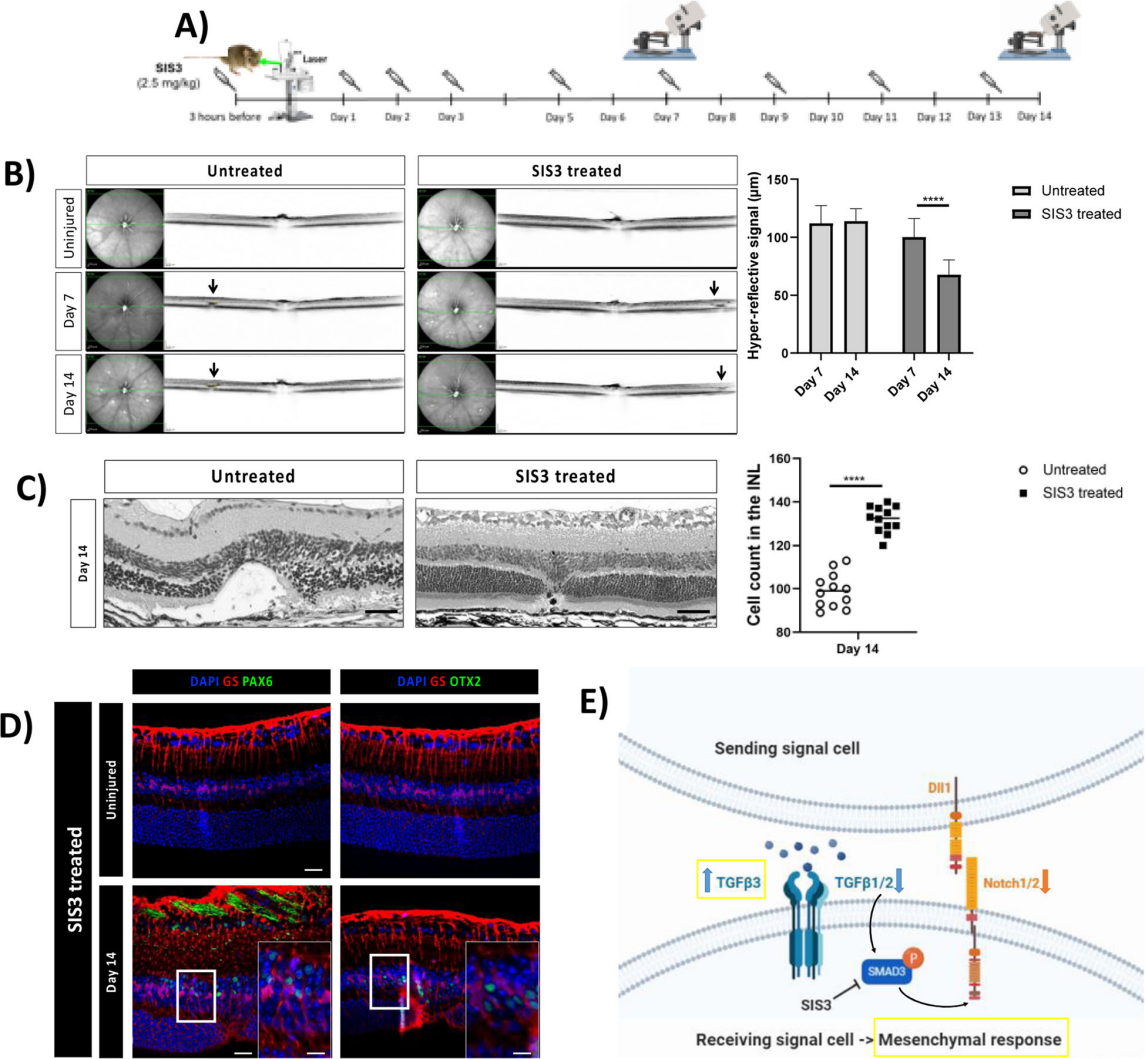
Pharmacological inhibition of p-Smad3 (SIS3) diminishes retinal damage. (**A)** SIS3 or PBS injections performed at 3 h before injury, daily during the first three days after injury, and then every other day until day 14. (**B)** IR (left) and OCT (right) images of the injury from a single animal at baseline, day 7 and 14. Arrows point to the central lesion depict the injury sites detected as hyper-reflective signal. Significant differences (****p < 0.001) between different time points were determined by two-tailed t-test (*n* = 12). (**C)** H&E-stained images of untreated and SIS3-treated retinas at day 14 after injury. Analyzed length was 100 μm, corresponding to the induced-laser burn. Significant differences in structural changes (****p < 0.0001) between untreated and SIS3 treated groups were determined by two-tailed t-test (*n* = 12). (**D)** Detection of PAX6 and OTX2 in GS^+^MCs in untreated and SIS3-treated mice. Shown are representative sections for GS (red) and PAX6 or OTX2 (green). (**E)** Schematic summary of molecular outcomes of SIS3 treatment in murine MCs. INL, inner nuclear layer; ONL, outer nuclear layer. Scale bar of the images equals 50 μm, while in the inserts corresponding to 150 μm

TGFβ and Notch regulate similar physiological as well as pathological processes. They also show frequent crosstalk in different tissues and organs [10]. Based on the cellular context, TGFβ/Notch can antagonize or synergize each other in a Smad3-dependent manner [36]. However, mechanisms of TGFβ/Notch interplay are unknown during reactive gliosis. Thus, we studied whether it was mediated by Smad3 and the effect on reactive gliosis. Mice were treated with either SIS3, a specific Smad3 inhibitor, or PBS by intraperitoneal injection either at 3 h before injury (baseline), at day 2 (MC-cycle arrest) or at day 6 (MC-ET). One day after injection, mice were euthanized (Day 1, 3 and 7, respectively) and the expression pattern were compared to each other at the different timepoints (Fig. 8A). To test the efficiency of SIS3 during injury response, we analyzed p-Smad3 expression in murine MCs (GS^+^) by immunofluorescence in both SIS3 and untreated groups (Fig. S6A-G). In accordance with previous studies [37], SIS3 treatment for 24 h inhibited Smad3 phosphorylation (Fig. S6A-C, G). In control animals (Untreated), p-Smad3 was detectable from day 1 (Fig. S6D, G) with the maximum signal at day 3 (Fig. S6E, G). The effects of SIS3 on MC injury response was investigated by immunofluorescence of TGFβ1/2/3 and E- as well as N-cadherin in GS^+^ MCs (Fig. 8B-H). Among TGFβ isoforms, only TGFβ3 was upregulated in response to injury in SIS3 treated mice at every timepoint (Days 1, 3, and 7; Fig. 8D.i-D.iii, E). Together with TGFβ3 upregulation, we detected downregulation of E-cadherin and an upregulation of N-cadherin (Fig. 8F-H), suggesting that SIS3-induced-TGFβ3 may trigger a mesenchymal response in murine MCs. To study the beneficial effect of SIS3-treatment throughout reactive gliosis, one group of mice was treated with SIS3 three h before injury, daily during the first 3 days after injury, and then every other day until day 14 (Fig. 9A) whereas the control group was injected with PBS (Untreated). The extent of the injured area was investigated by SD-OCT at days 7 and 14 as well as in H&E stained sections at day 14 (Fig. 9B-C) and compared to each other. At day 7 we identified the injured area as a compact dome-shaped hyper-reflective signal located between the RPE and the outer plexiform layer (OPL) in both groups (Fig. 9B). No significant difference in the hyper-reflective signal was detected between days 7 and 14 in the control group (Untreated; Fig. 9B). Interestingly, SIS3 treated mice showed a significant reduction in the injured area to ~ 68 μm at day 14 (Fig. 9B), which suggested that the pathological gliotic changes were attenuated by SIS3. Relevant differences were detected by morphometric analysis at day 14 (Fig. 9C). We observed cavity formation in the ONL and a thinning of the INL due to loss of nuclei within the damaged area in the untreated mice. Instead, the number of nuclei in the INL was doubled only in the injured area of SIS3-treated mice, possibly due to MC generation of progenitors that migrate to the ONL to restore it (Fig. 9C). Therefore, to investigate whether MC are de-differentiating after SIS3 treatment, we investigated by immunofluorescence progenitor markers, PAX6 and OTX2, in GS^+^ MCs in the injured and contralateral uninjured eyes at day 14 (Fig. 9D). Both PAX6 and OTX2 were detected in GS^+^ MCs in the injured eye only, suggesting that SIS3 may favor reprogramming of murine MCs into progenitor cells upon injury.

## Discussion

Through cross-species comparison, we determined the importance of TGFβ/Notch during MC injury response. We revealed that TGFβ/Notch interplay, in a Smad3-dependent manner, triggers MC-cycle arrest resulting in unsuccessful reprogramming during reactive gliosis in mice. Inhibiting Smad3 boost the limited regenerative potential of murine MCs. Moreover, our findings suggest a MC shift towards an epithelial lineage (MC-ET) during reactive gliosis in mammals, shedding new light into the remodeling mechanism of retinal degeneration.

Upon injury, reactive gliosis includes distinctive morphological and molecular alterations in MCs [38]. Different MC reactivity types are known. Whereas non-specific responses (upregulation of GFAP and phospho-Erk1/2) are independent of the causative stimulus, depend specific gliotic responses (upregulation of GS) on the respective pathological condition [21]. We detected a temporary expression of both non-specific and specific markers in zebrafish (Fig. 1A, C, E), which suggests a transient gliosis and its regression is concomitant with the regeneration of the photoreceptor layer. In contrast, MC gliosis in mice persists until the last timepoint investigated (Day 14; Fig. 1B, D, E). In line with previous studies [39], we showed that our injury model effectively simulates a chronic gliotic response. However, it is unclear how chronic MC reactivity in mammals exacerbates injury response leading to glial scar formation. Another important feature of MC reactivity is their exit from quiescence in response to injury [40]. We evaluated MC re-entering the cell cycle using PCNA in both animal models (Fig. 1F-H). Though we detected the simultaneous re-entry into the cell-cycle in both zebrafish and murine MCs (Day 3), in zebrafish the signal returned to baseline in the restored retina, which supports the hypothesis of a transient reactive gliosis. However, murine MCs appeared proliferative throughout the experiment. This is in contrast to the current knowledge that MC cell cycle rarely reaches S-phase. Indeed, analysis of the cellular DNA content reveled an abnormal accumulation of murine MC in G2/M-phase (Fig. 2A, B), suggesting arrested re-entry into mitosis. Mitosis is a highly dynamic process and its failure can activate DDR [41]. One of the most accepted chromatin modification markers linked to DDR is γH2A.X [42]. Its quantification in MCs showed double-strand breaks (DSBs) during injury response in mice only (Fig. S1 D-G). The activation of DDR induces the growth arrest of damaged cells and allows the DNA repair to mend the damaged DNA [43]. Therefore, we investigate H2A.Z, involved in the reorganization of chromatin architecture and in the assembly of a chromatin template [44]. We detect an activation of the DNA repair mechanisms by H2A.Z upregulation (Fig. S1 K-N), showing that murine MCs can protect the integrity of their genome from DSBs. Once repair is over, MCs should exit the checkpoints and restore retinal functionality. Instead, MCs form a gliotic scar impeding retinal regeneration in mammals. Recently, Peñalosa-Ruiz et al. [45] demonstrated that DDR is resolved by somatic cell reprogramming. Silencing of somatic genes during DDR is concurrent with the acquisition of epithelial features [46] and most of the cells are trapped in such stage. Only a minimal proportion progresses toward pluripotency [47]. In line with this idea, we identify the initial N-cadherin expression in both animal models (Fig. 3F-H), suggesting the possibility of MCs to behave as progenitor/stem cells. However, only murine MCs undergo epithelial-like changes (E-cadherin at day 7; Fig. 3A-E). The unsuccessful reprogramming into progenitors during chronic reactive gliosis illustrates that murine MCs are not able to ensure a proper segregation of the duplicated genome during injury response leading to arrested re-entry into mitosis. That may lead to DSBs with the ensuing acquisition of epithelial features by MCs during glial scar formation. MC-ET can be promoted by a variety of signals, such as TGFβ, known to mediate MET [48]. However, pathways are not independent from each other, and they can interact to form complex networks. Possibly, due to its involvement during various cellular processes (e.g., proliferation, differentiation, and apoptosis), TGFβ pathway interacts with other pathways during MET [49]. TGFβ cytostatic response, characterized by DDR, requires Notch. Furthermore, Notch controls transition through late stages of the cell-cycle and its timing is crucial for determining the decision of precursors to progress to a neural fate [50]. Recently, we investigated TGFβ family members and the downstream signaling mediators that are associated with repair mechanisms in zebrafish and in mice. We showed that TGFβ3 promotes regeneration via TGFβ canonical pathway in zebrafish MCs [6]. Whereas, in mice, TGFβ1/2 evokes the activation of the non-canonical TGFβ pathway during scar formation. Here, we identified the activation of Notch pathway via Notch1/2 in response to injury in mice only (Fig. 4A-D). The simultaneous activation of TGFβ and Notch in murine MCs suggests their combined action during chronic reactive gliosis. Despite of many studies, significant questions arise regarding the relevance of murine injury model to human retinal degeneration. Upon injury, murine MCs undergo chronic reactive gliosis, pathological feature observed in most human neurodegenerations [51]. To determine the significance of our findings, we subdivided human retinas in samples with healthy cuboidal RPE, and retinas with drusen (Fig. 5A-C). We confirmed the gliotic response occurring together with the acquisition of an epithelial phenotype by human reactive MCs solely in retinas presenting drusen (Fig. 5D-J). Furthermore, we detected TGFβ1/Notch2 expressions in human gliotic MCs only (Drusen pos: Fig. 5K-O). Altogether, these data indicate a direct link between MC-ET and TGFβ/Notch during chronic reactive gliosis in human, as in mice during injury response. We systematically investigated the impact of arrested re-entry into mitosis on retinal regeneration in zebrafish, and either TGFβ or Notch inhibition on reactive gliosis in mice. Palbociclib was used to induce cell-cycle arrest in order to investigate if that is sufficient to stimulate MC-ET in zebrafish (Days 4, 5 and 6; Fig. S3 A-D). Although we showed DDR upon this treatment by HA2.X upregulation, we did not detect H2A.Z, involved in DNA repair mechanism (Fig. S3 C-D). These data suggest that the induced cell-cycle arrest in zebrafish as well as arrested re-entry into mitosis in mouse may be the trigger for DDR. However, it will not lead to a proper assembly of a chromatin template, which is an efficient substrate for the DSB repair machinery (Fig. S3 D). Additionally, we found a consistent association between DDR in MCs and a fibrotic-like outcome (CTGF), at the expense of MC mesenchymal (neural) potential (N-cadherin; Fig. S4 A-B). CTGF is linked to MET in the pathogenesis of fibrotic diseases (e.g., renal, myocardial, pulmonary fibrosis) [52]. Unfortunately, we could treat zebrafish for 24 h only as further addition of the drug led to behavioral alterations (e.g., changes in activity, schooling behavior, social interaction) and, thus, the fish had to be excluded from the study. Nevertheless, these data allowed us to discriminate the effect of DNA damage from its repair and, therewith, to propose MC-ET as a repair mechanism following cell-cycle arrest. MET-associated fibrosis is regulated by various molecular mechanisms, among which Notch and TGFβ are important regulators [53, 54]. After treatment, we found an upregulation of Notch1/2 in zebrafish MCs (Fig. S4 C-D), suggesting that DDR may be linked to Notch1/2 [55]. Contradicting each other, we detected the expression of both TGFβ1, which endorses fibrosis, and the anti-fibrotic TGFβ3 [56]. Therefore, we deduced how palbociclib may induce DDR enough to show the link between Notch1/2 and TGFβ1 during scar formation. Contrariwise, palbociclib carcinogenicity limited the possibility to verify the long-term effect of DDR, possibly impeding TGFβ3 downregulation and the detection of zebrafish MC-ET. Pirfenidone has been already employed in clinical trials for the treatment of fibrosis [57] and displayed the potential to revert TGFβ-induced MET [58]. Thus, we treated mice during early injury response (baseline to Day 1), MC-cycle arrest (Day 2–3), and MC-ET (Day 6–7; Fig. S5 A-I). TGFβ inhibition hindered both E- and N-cadherin and Notch1/2 expressions in mice trapping MCs into quiescence even after injury (Fig. S5 E-H). However, targeting TGFβ may affect other physiological mechanisms (e.g., wound healing, immune regulation), owing its pleiotropic nature. Pirfenidone revealed that TGFβ may act upstream of Notch. Notch pathway is essential for cell fate during embryogenesis, stem cell self-renewal, and tissue differentiation [59]. Notch is also critical for the pathogenesis of fibrotic diseases and it is involved in the induction of MET [9, 54]. Given that we were determined to preserve TGFβ and prevent MC-ET-associated fibrosis, we used a pharmacological inhibitor of γ-secretase (DAPT) to block Notch action [60]. Mice were treated for 24 h during early injury response, MC cell-cycle arrest, and MC-ET (Fig. 6A-I). DAPT boosted N-cadherin expression in murine MCs at every stage of the injury response (Fig. 6D). Concomitant with MC mesenchymal response, DAPT promoted anti-fibrotic TGFβ3, but kept the expression of pro-fibrotic TGF1/2 (Fig. 7A-E). Therewith, we revealed a potential differentiation-inducing effect of DAPT. Transient exposure with DAPT may drive MCs towards restoring the retina. Prolonged DAPT treatment could be detrimental because it might interfere with MC reactivity, which is regulated by Notch signaling. However, no study has described the timeline during which Notch has to be inactive to cause neural de-differentiation. Short-term DAPT treatment (24 h) confirmed the link between TGFβ/Notch. However, pro-fibrotic TGFβ response seems independent to Notch inhibition (Fig. 7A-B). Both inhibitions of either TGFβ or Notch were ineffective to induce further improvements during injury response in mice. Ultimately, we hypothesize that the combined action of TGFβ/Notch may mediate MC-ET. Mechanistically, TGFβ/Notch interplay can occur at multiple levels [10]. TGFβ cooperates with Notch in a Smad3-dependent manner [36] and both synergistic as well as antagonistic effects of TGFβ/Notch interplay have been reported in various cellular contexts [61]. However, outcomes of their interaction during chronic reactive gliosis in mammals have not been reported to date. In line with this, we observed p-Smad3 signal in mice independently from the activation of the non-canonical TGFβ signaling after injury [6]. Furthermore, most of TGFβ pro-fibrotic activities are mediated by Smad3 and genetic deletion of Smad3 interferes with TGFβ-mediated MET in fibrotic diseases [62]. We presumed that blocking Smad3 may attenuate TGFβ/Notch interplay and interfere with glial scar formation in mice. Small molecule inhibitors of Smad3, might have an incredible clinical potential in the treatment of MET-associated fibrotic diseases, as chronic reactive gliosis. Therefore, we suppressed p-Smad3 using SIS3 during early injury response, MC cell-cycle arrest, and MC-ET in mice (Figs. 8 and 9). Once more, TGFβ3 hand-in-hand with N-Cadherin favored a mesenchymal response at the expense of MC-ET via TGFβ1/2 after injury (Fig. 8B-H). Transient treatment (24 h) with SIS3 ameliorated MC injury response at every stage investigated (Fig. 8A-H). Based on this promising data, we extend SIS3 treatment to until day 14 (Fig. 9A-D). SIS3 treated mice displayed a significant reduction of the glial scar unlike control group (Fig. 9B-C). The reduction of the glial scar was associated with MC de-differentiation at day 14, showing that, after SIS3 treatment, murine MCs are capable to exit their quiescence state, critical step toward regeneration. In our study, SIS3 showed the potential to modulate MC-ET-associated fibrosis, but also reduced the glial scar in vivo by stimulating MC de-differentiation. Furthermore, SIS3 treatment reveals the possible communication between TGFβ1/2 with Notch1/2 and their combined action might promote the transition from a mesenchymal to an epithelial phenotype in mammalian MCs (MC-ET) after injury. Thus, Smad3 seems a promising target to ameliorate the detrimental effects of chronic reactive gliosis, such as glial scar formation known as a physical barrier of retinal regeneration in mammals [63].

## Conclusions

Summarizing our previous and current findings, DDR may stimulate MC pro-fibrotic response and TGFβ interplay with Notch via Smad3, culminating in the acquisition of epithelial features in mammalian MCs (MC-ET). Furthermore, we propose that blocking the combined action of TGFβ/Notch unlocks MC mesenchymal response via TGFβ3. Our findings open new avenues for research aimed at developing therapeutic strategies on endogenous repair of the retina.

## Supplementary Information

**Additional file 1: Figure S1.** DDR response and repair in zebrafish and murine MCs in response to injury. (A-G) Analysis of MC DDR response in zebrafish and mice at the baseline (Uninjured) and at different time points after injury (Day 1, 3 and 7). Detection of H2A.X in GS^+^ MCs after laser induction in zebrafish (A-C) and mice (D-F). Shown are representative retinal sections stained for GS (red) and H2A.X (green). Zoomed-in view of murine GS^+^/H2A.X^+^ cells of the area defined by a blue frame at Day 3 (E.i-E.iv). White arrowheads mark double-positive cells. (G) Histograms illustrating the mean ± SD of the number of H2A.X^+^ cells normalized by the total number of GS^+^ cells expressed in percentage. Significant differences (****p < 0.0001) between uninjured and injured retinas were determined by using a post-hoc Bonferroni one-way ANOVA test (*n* = 12). (H-N) Analysis of MC DNA repair in zebrafish and mice at the baseline (Uninjured) and at different time points after injury (Day 1, 3 and 7). Detection of H2A.Z in GS^+^ MCs after laser induction in zebrafish (H-J) and mice (K-M). Shown are representative retinal sections stained for GS (red) and H2A.Z (green). Zoomed-in view of murine GS^+^/H2A.Z^+^ cells of the area defined by a blue frame at days 1 and 3 (K.i-K.iv, L.i-L.iv). White arrowheads mark double-positive cells. (N) Histograms illustrating the mean ± SD of the number of H2A.Z^+^ cells normalized by the total number of GS^+^ cells expressed in percentage. Significant differences (****p < 0.0001) between uninjured and injured retinas were determined by using a post-hoc Bonferroni one-way ANOVA test (n = 12). INL, inner nuclear layer; ONL, outer nuclear layer. Scale bar of all images equals 50 μm, while in the zoom-in view corresponding to 150 μm. **Figure S2.** Ingenuity pathway analysis to investigate changes in gene expression during MC-ET. Data are expressed as fold-changes or Log ratio compared to negative controls (cycling MCs from uninjured retinas). **Figure S3.** Efficiency of palbociclib pharmacological treatment in zebrafish. (A-D) Evaluation of pharmacologically induced G2/M arrest using palbociclib in zebrafish MC at different time points after injury (Day 1, 3 and 7). Zebrafish were immersed in palbociclib water (tubes; 2 μM final concentration in tank water) at different time points (Day 4, 5 and 6) after injury induction. One day after treatment, zebrafish were euthanized (orange arrows; Day 5, 6 and 7). Detection of PCNA (A.i-A.iii), p-H3 (B.i-B.iii), H2A.X (C.i-C.iii) and H2A.Z (D.i-D.iii) in GS^+^ MCs after laser induction in zebrafish. Shown are representative retinal sections stained for GS (red) and PCNA, p-H3, H2A.X and H2A.Z (green). (A.iv, B.iv, C.iv, D.iv) Histograms illustrating the mean ± SD of the number of PCNA^+^, p-H3^+^, H2A.X^+^ and H2A.Z^+^ cells normalized by the total number of GS^+^ cells expressed in percentage. INL, inner nuclear layer; ONL, outer nuclear layer. Scale bar of all images equals 50 μm. **Figure S4.** Pharmacological G2/M arrest (palbociclib). (A-B) Analysis of MC phenotype in palbociclib treated zebrafish at different time points (Day 5, 6 and 7). Detection of N-Cadherin (A.i-A.iii) and CTGF (B.i-B.iii) in GS^+^ MCs. Shown are representative sections stained for GS (red) and N-Cadherin/CTGF (green). (A.iv, B.iv) Histograms illustrating the mean ± SD of the number of N-Cadherin^+^ and CTGF^+^ cells normalized by the total number of GS^+^ cells expressed in percentage (n = 12). (C-D) Analysis of Notch isorforms during MC injury response in palbociclib treated zebrafish at different time points (Day 5, 6 and 7). Detection of Notch1 (C.i-C.iii) and Notch2 (D.i-D.iii) in GS^+^ MCs. Shown are representative sections stained for GS (red) and Notch1/2 (green). (C.iv, D.iv) Histograms illustrating the mean ± SD of the number of Notch1^+^ and Notch2^+^ cells normalized by the total number of GS^+^ cells expressed in percentage (n = 12). (E-H) Analysis of TGFβ pathway during MC injury response in palbociclib treated zebrafish at different time points (Day 5, 6 and 7). Detection of TGFβ1 (E.i-E.iii), TGFβ2 (F.i-F.iii), TGFβ3 (G.i-G.iii) and pSmad3 (H.i-H.iii) in GS^+^ MCs. Shown are representative sections stained for GS (red), TGFβ1/2/3 and pSmad3 (green). (E.iv, F.iv, G.iv, H.iv) Histograms illustrating the mean ± SD of the number of TGFβ1^+^, TGFβ2^+^, TGFβ3^+^ and pSmad3^+^ cells normalized by the total number of GS^+^ cells expressed in percentage (n = 12). INL, inner nuclear layer; ONL, outer nuclear layer. Scale bar of all images equals 50 μm. (I) Schematic summary of molecular outcomes of palbociclib treatment in zebrafish MCs. **Figure S5.** Pharmacological TGFβ inhibition (pirfenidone). Mice were treated with pirfenidone (50 mg/kg) by intraperitoneal injection either at 3 h before injury, at day 2 or at day 6 (syringes) and euthanized 24 h after injection (orange arrows; Day 1, 3, 7). (A-D) Analysis of TGFβ pathway during MC injury response in pirfenidone treated mice at different time points (Day 1, 3 and 7). Detection of TGFβ1 (A.i-A.iii), TGFβ2 (B.i-B.iii), TGFβ3 (C.i-C.iii) and pSmad3 (D.i-D.iii) in GS^+^ MCs. Shown are representative sections stained for GS (red), TGFβ1/2/3 and pSmad3 (green). (E-F) Analysis of MC phenotype during injury response in pirfenidone treated mice at different time points (Day 1, 3 and 7). Detection of E-Cadherin (E.i-E.iii) and N-Cadherin (F.i-F.iii) in GS^+^ MCs. Shown are representative sections stained for GS (red), E−/N-Cadherin (green). (G-H) Analysis of Notch isoforms during MC injury response in pirfenidone treated mice at different time points (Day 1, 3 and 7). Detection of Notch1 (G.i-G.iii) and Notch2 (H.i-H.iii) in GS^+^ MCs. Shown are representative sections stained for GS (red), Notch1/2 (green). INL, inner nuclear layer; ONL, outer nuclear layer. Scale bar of all images equals 50 μm. (I) Schematic summary of molecular outcomes of pirfenidone treatment in murine MCs. **Figure S6.** Efficiency of SIS3 pharmacological treatment in mice. (A-G) Analysis of pSmad3 inhibition using SIS3 in murine MC at different time points after injury (Day 1, 3 and 7). Detection of pSmad3 in GS^+^ MCs after laser induction in SIS3 treated (A-C) and untreated mice (D-F). Shown are representative retinal sections stained for GS (red) and pSmad3 (green). (G) Histogram illustrating the mean ± SD of the number of pSmad3^+^ cells normalized by the total number of GS^+^ cells expressed in percentage. INL, inner nuclear layer; ONL, outer nuclear layer. Scale bar of all images equals 50 μm.

## Data Availability

RNA-Sequencing data have been deposited in the Gene Expression Omnibus database with accession numbers: GSE132141 (zebrafish) and GSE132140 (mouse). Additional data that support the findings of this study can be found in Supplementary Information.

## Abbreviations

DAPT: (2*S*)-*N*-[(3,5-Difluorophenyl)acetyl]-L-alanyl-2-phenyl] glycine 1,1-dimethylethyl ester
DDR: DNA damage response
Erk: Extracellular signal-regulated kinases
GFAP: Glial fibrillary acidic protein
GFP: Green fluorescent protein
GS: Glutamine synthetase
MC: Müller cells
MC-ET: Müller cell-epithelial-transition
MET: Mesenchymal-epithelial-transition
OTX2: Orthodenticle homeobox 2
PAX6: Paired box protein 6
Rlbp 1: Retinaldehyde-binding protein 1
SD-OCT: Spectral domain-optical coherence tomography
SIS3: Specific inhibitor of Smad3
Smad3: Mothers against decapentaplegic homolog 3
TGF: Transforming growth factor
