# Supplementary material for: The TGFβ/Notch axis facilitates Müller cell-to-epithelial transition to ultimately form a chronic glial scar
Source: Mol Neurodegener. 2021 Sep 30;16:69. doi: 10.1186/s13024-021-00482-z (PMC8482586; doi:10.1186/s13024-021-00482-z)
Supplement: Supplementary file 1 — Additional file 1: Figure S1. DDR response and repair in zebrafish and murine MCs in response to injury. (A-G) Analysis of MC DDR response in zebrafish and mice at the baseline (Uninjured) and at different time points after injury (Day 1, 3 and 7). Detection of H2A.X in GS+ MCs after laser induction in zebrafish (A-C) and mice (D-F). Shown are representative retinal sections stained for GS (red) and H2A.X (green). Zoomed-in view of murine GS+/H2A.X+ cells of the area defined by a blue frame at Day 3 (E.i-E.iv). White arrowheads mark double-positive cells. (G) Histograms illustrating the mean ± SD of the number of H2A.X+ cells normalized by the total number of GS+ cells expressed in percentage. Significant differences (****p < 0.0001) between uninjured and injured retinas were determined by using a post-hoc Bonferroni one-way ANOVA test (n = 12). (H-N) Analysis of MC DNA repair in zebrafish and mice at the baseline (Uninjured) and at different time points after injury (Day 1, 3 and 7). Detection of H2A.Z in GS+ MCs after laser induction in zebrafish (H-J) and mice (K-M). Shown are representative retinal sections stained for GS (red) and H2A.Z (green). Zoomed-in view of murine GS+/H2A.Z+ cells of the area defined by a blue frame at days 1 and 3 (K.i-K.iv, L.i-L.iv). White arrowheads mark double-positive cells. (N) Histograms illustrating the mean ± SD of the number of H2A.Z+ cells normalized by the total number of GS+ cells expressed in percentage. Significant differences (****p < 0.0001) between uninjured and injured retinas were determined by using a post-hoc Bonferroni one-way ANOVA test (n = 12). INL, inner nuclear layer; ONL, outer nuclear layer. Scale bar of all images equals 50 μm, while in the zoom-in view corresponding to 150 μm. Figure S2. Ingenuity pathway analysis to investigate changes in gene expression during MC-ET. Data are expressed as fold-changes or Log ratio compared to negative controls (cycling MCs from uninjured retinas). Figure S3. Efficiency o [file 13024_2021_482_MOESM1_ESM.pdf]

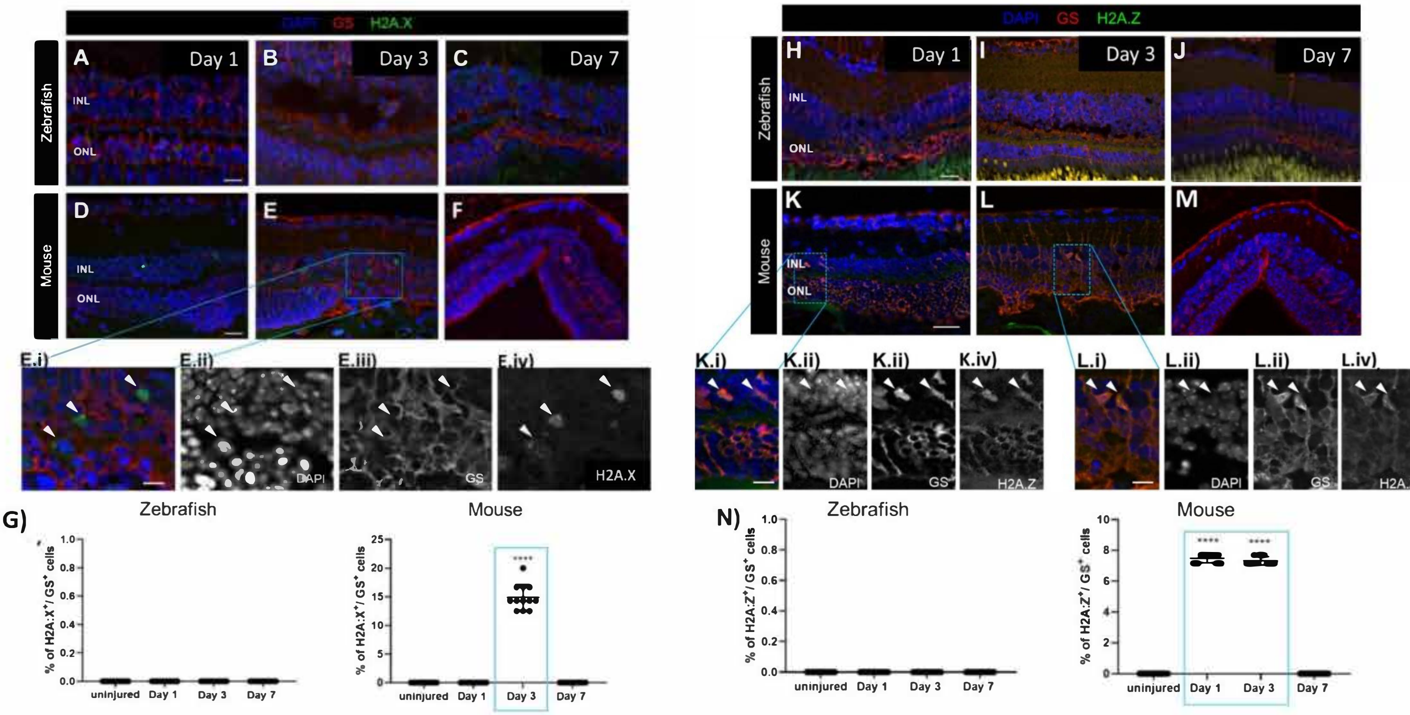

Figure S1: DDR response and repair in zebrafish and murine MCs in response to injury. (A-G) Analysis of MC DDR response in zebrafish and mice at the baseline (Uninjured) and at different time points after injury (Day 1, 3 and 7). Detection of H2A.X in GS+ MCs after laser induction in zebrafish (A-C) and mice (D-F). Shown are representative retinal sections stained for GS (red) and H2A.X (green). Zoomed-in view of murine GS+/H2A.X+ cells of the area defined by a blue frame at Day 3 (E.i-E.iv). White arrowheads mark double-positive cells. (G) Histograms illustrating the mean ± SD of the number of H2A.X+ cells normalized by the total number of GS+ cells expressed in percentage. Significant differences (****$p<0.0001$) between uninjured and injured retinas were determined by using a post-hoc Bonferroni one-way ANOVA test (n=12). (H-N) Analysis of MC DNA repair in zebrafish and mice at the baseline (Uninjured) and at different time points after injury (Day 1, 3 and 7). Detection of H2A.Z in GS+ MCs after laser induction in zebrafish (H-J) and mice (K-M). Shown are representative retinal sections stained for GS (red) and H2A.Z (green). Zoomed-in view of murine GS+/H2A.Z+ cells of the area defined by a blue frame at days 1 and 3 (K.i-K.iv, L.i-L.iv). White arrowheads mark double-positive cells. (N) Histograms illustrating the mean ± SD of the number of H2A.Z+ cells normalized by the total number of GS+ cells expressed in percentage. Significant differences (****$p<0.0001$) between uninjured and injured retinas were determined by using a post-hoc Bonferroni one-way ANOVA test (n=12). INL, inner nuclear layer; ONL, outer nuclear layer. Scale bar of all images equals 50 µm, while in the zoom-in view corresponding to 150 µm.

Mouse

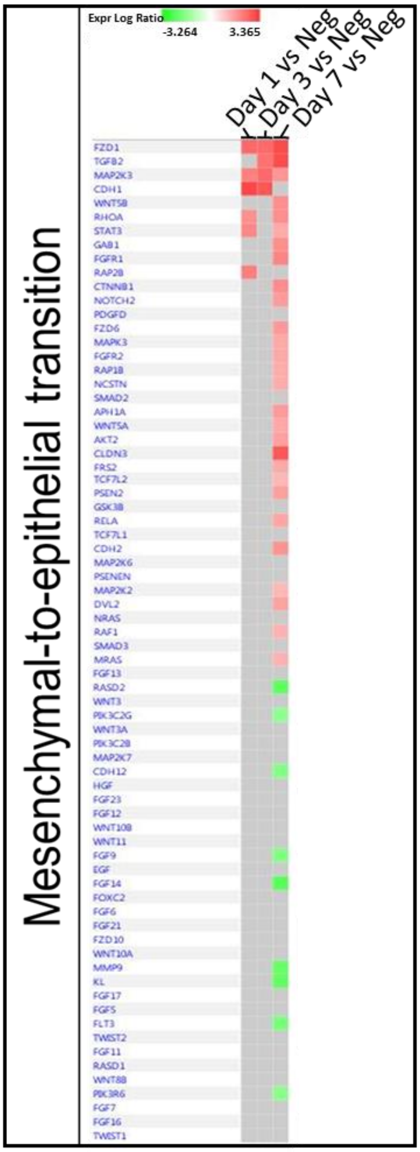

Figure S2: Ingenuity pathway analysis to investigate changes in gene expression during MC-ET. Data are expressed as fold-changes or Log ratio compared to negative controls (cycling MCs from uninjured retinas).

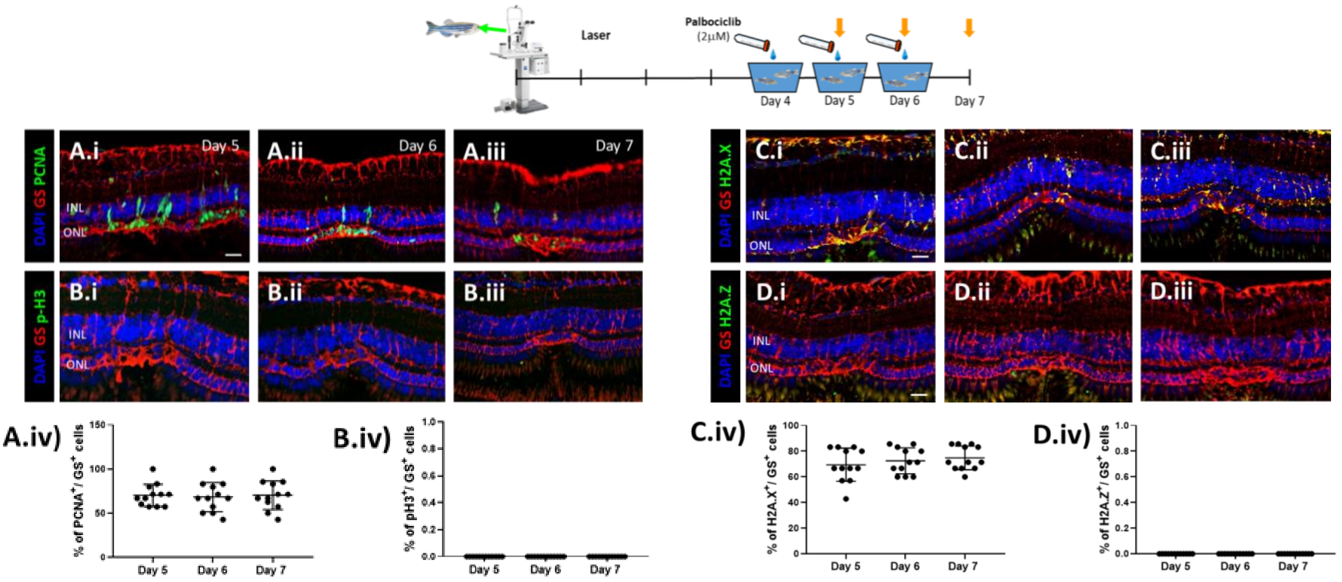

Figure S3: Efficiency of palbociclib pharmacological treatment in zebrafish. (A-D) Evaluation of pharmacologically induced G2/M arrest using palbociclib in zebrafish MC at different time points after injury (Day 1, 3 and 7). Zebrafish were immersed in palbociclib water (tubes; 2 µM final concentration in tank water) at different time points (Day 4, 5 and 6) after injury induction. One day after treatment, zebrafish were euthanized (orange arrows; Day 5, 6 and 7). Detection of PCNA (A.i-A.iii), p-H3 (B.i-B.iii), H2A.X (C.i-C.iii) and H2A.Z (D.i-D.iii) in GS+ MCs after laser induction in zebrafish. Shown are representative retinal sections stained for GS (red) and PCNA, p-H3, H2A.X and H2A.Z (green). (A.iv, B.iv, C.iv, D.iv) Histograms illustrating the mean ± SD of the number of PCNA+, p-H3+, H2A.X+ and H2A.Z+ cells normalized by the total number of GS+ cells expressed in percentage. INL, inner nuclear layer; ONL, outer nuclear layer. Scale bar of all images equals 50 µm.

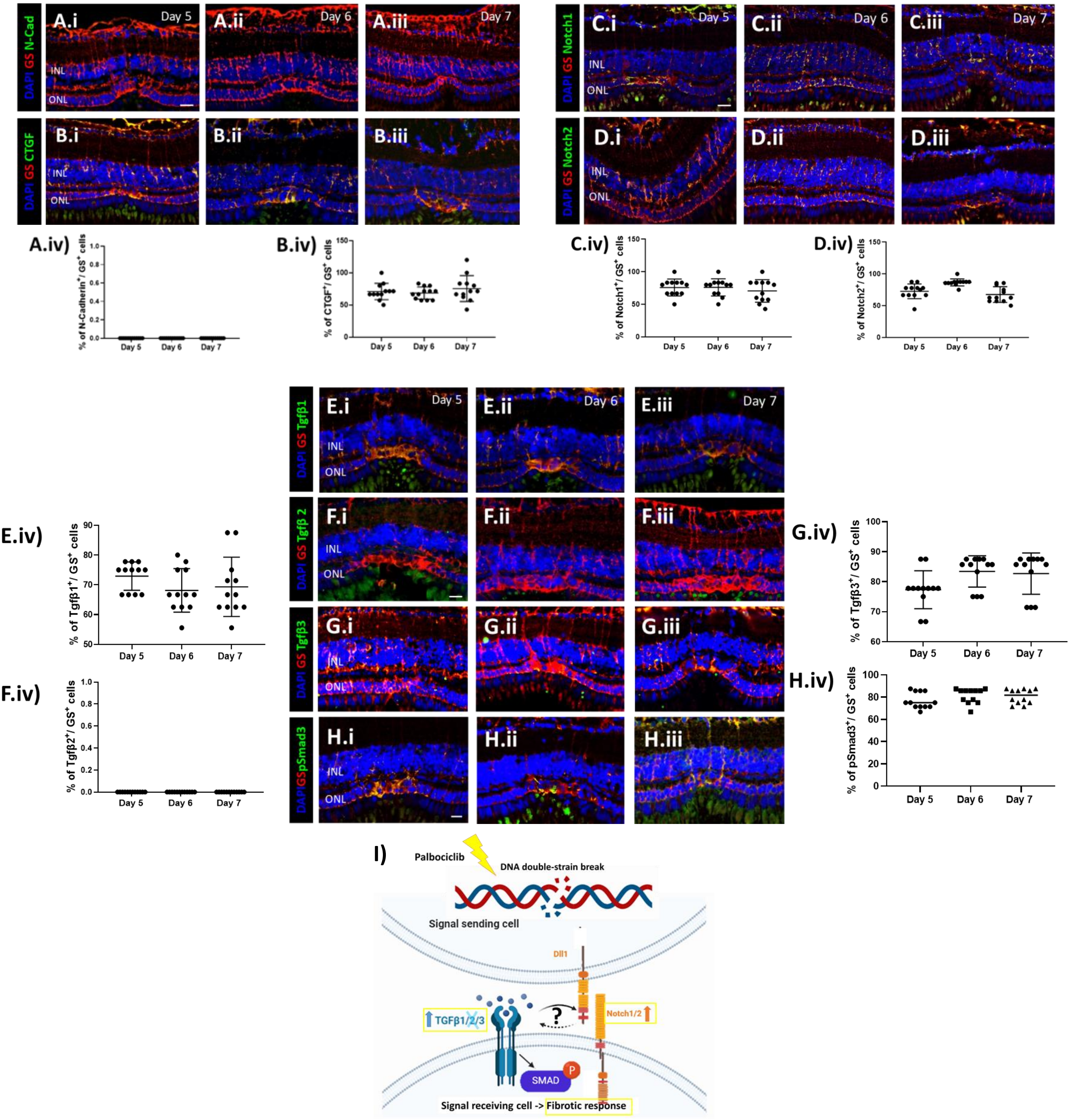

Figure S4: Pharmacological G2/M arrest. (A-B) Analysis of MC phenotype in palbociclib treated zebrafish at different time points (Day 5, 6 and 7). Detection of N-Cadherin (A.i-A.iii) and CTGF (B.i-B.iii) in GS+ MCs. Shown are representative sections stained for GS (red) and N-Cadherin/CTGF (green). (A.iv, B.iv) Histograms illustrating the mean ± SD of the number of N-Cadherin+ and CTGF+ cells normalized by the total number of GS+ cells expressed in percentage (n=12). (C-D) Analysis of Notch isorforms during MC injury response in palbociclib treated zebrafish at different time points (Day 5, 6 and 7). Detection of Notch1 (C.i-C.iii) and Notch2 (D.i-D.iii) in GS+ MCs. Shown are representative sections stained for GS (red) and Notch1/2 (green). (C.iv, D.iv) Histograms illustrating the mean ± SD of the number of Notch1+ and Notch2+ cells normalized by the total number of GS+ cells expressed in percentage (n=12). (E-H) Analysis of TGFβ pathway during MC injury response in palbociclib treated zebrafish at different time points (Day 5, 6 and 7). Detection of TGFβ1 (E.i-E.iii), TGFβ2 (F.i-F.iii), TGFβ3 (G.i-G.iii) and pSmad3 (H.i-H.iii) in GS+ MCs. Shown are representative sections stained for GS (red), TGFβ1/2/3 and pSmad3 (green). (E.iv, F.iv, G.iv, H.iv) Histograms illustrating the mean ± SD of the number of TGFβ1+, TGFβ2+, TGFβ3+ and pSmad3+ cells normalized by the total number of GS+ cells expressed in percentage (n=12). INL, inner nuclear layer; ONL, outer nuclear layer. Scale bar of all images equals 50 µm. (I) Schematic summary of molecular outcomes of palbociclib treatment in zebrafish MCs.

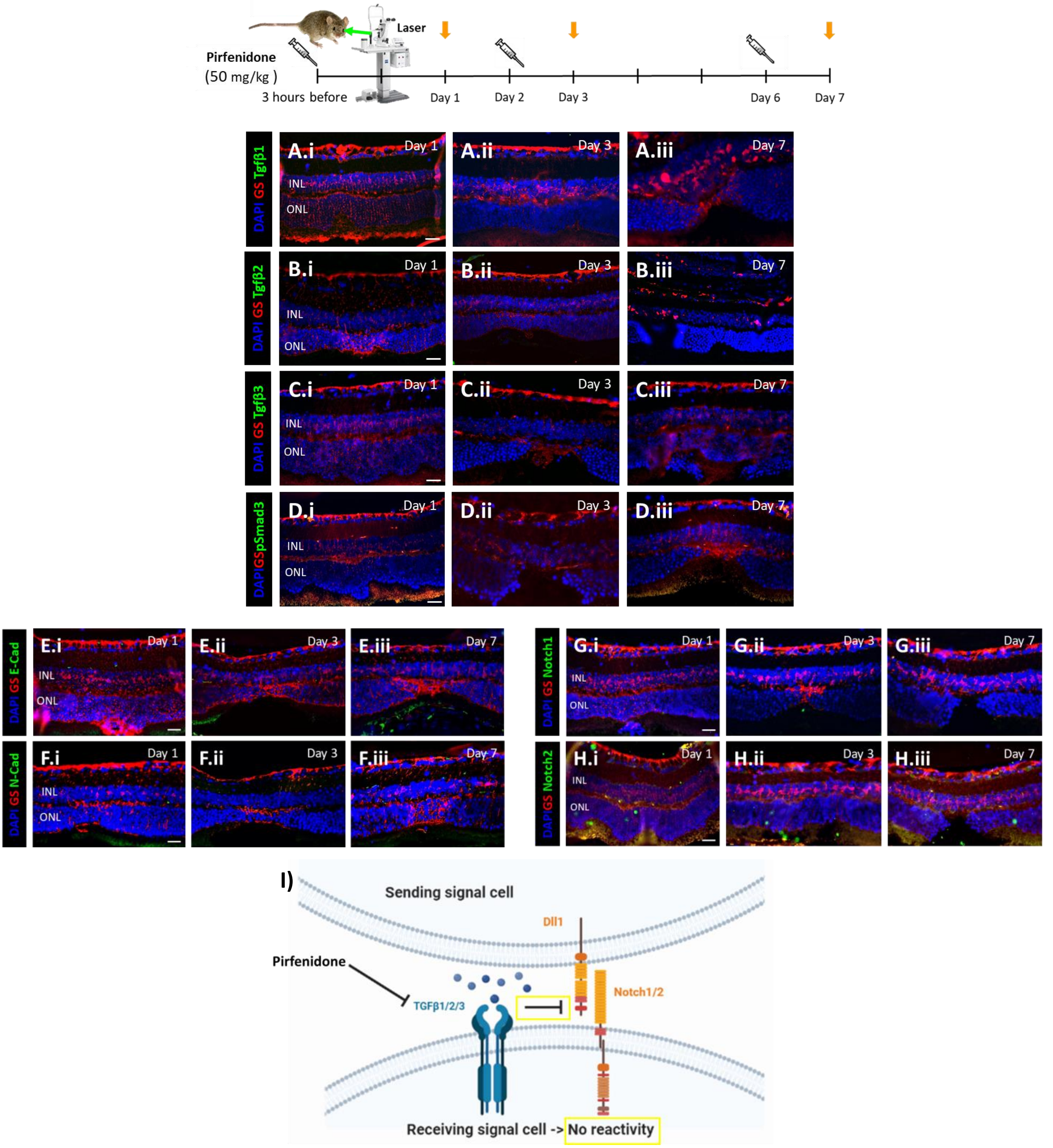

Figure S5: Pharmacological TGFβ inhibition. Mice were treated with pirfenidone (50 mg/kg) by intraperitoneal injection either at 3 h before injury, at day 2 or at day 6 (syringes) and euthanized 24 h after injection (orange arrows; Day 1, 3, 7). (A-D) Analysis of TGFβ pathway during MC injury response in pirfenidone treated mice at different time points (Day 1, 3 and 7). Detection of TGFβ1 (A.i-A.iii), TGFβ2 (B.i-B.iii), TGFβ3 (C.i-C.iii) and pSmad3 (D.i-D.iii) in GS+ MCs. Shown are representative sections stained for GS (red), TGFβ1/2/3 and pSmad3 (green). (E-F) Analysis of MC phenotype during injury response in pirfenidone treated mice at different time points (Day 1, 3 and 7). Detection of E-Cadherin (E.i-E.iii) and N-Cadherin (F.i-F.iii) in GS+ MCs. Shown are representative sections stained for GS (red), E-/N-Cadherin (green). (G-H) Analysis of Notch isoforms during MC injury response in pirfenidone treated mice at different time points (Day 1, 3 and 7). Detection of Notch1 (G.i-G.iii) and Notch2 (H.i-H.iii) in GS+ MCs. Shown are representative sections stained for GS (red), Notch1/2 (green). INL, inner nuclear layer; ONL, outer nuclear layer. Scale bar of all images equals 50 µm. (I) Schematic summary of molecular outcomes of pirfenidone treatment in murine MCs.

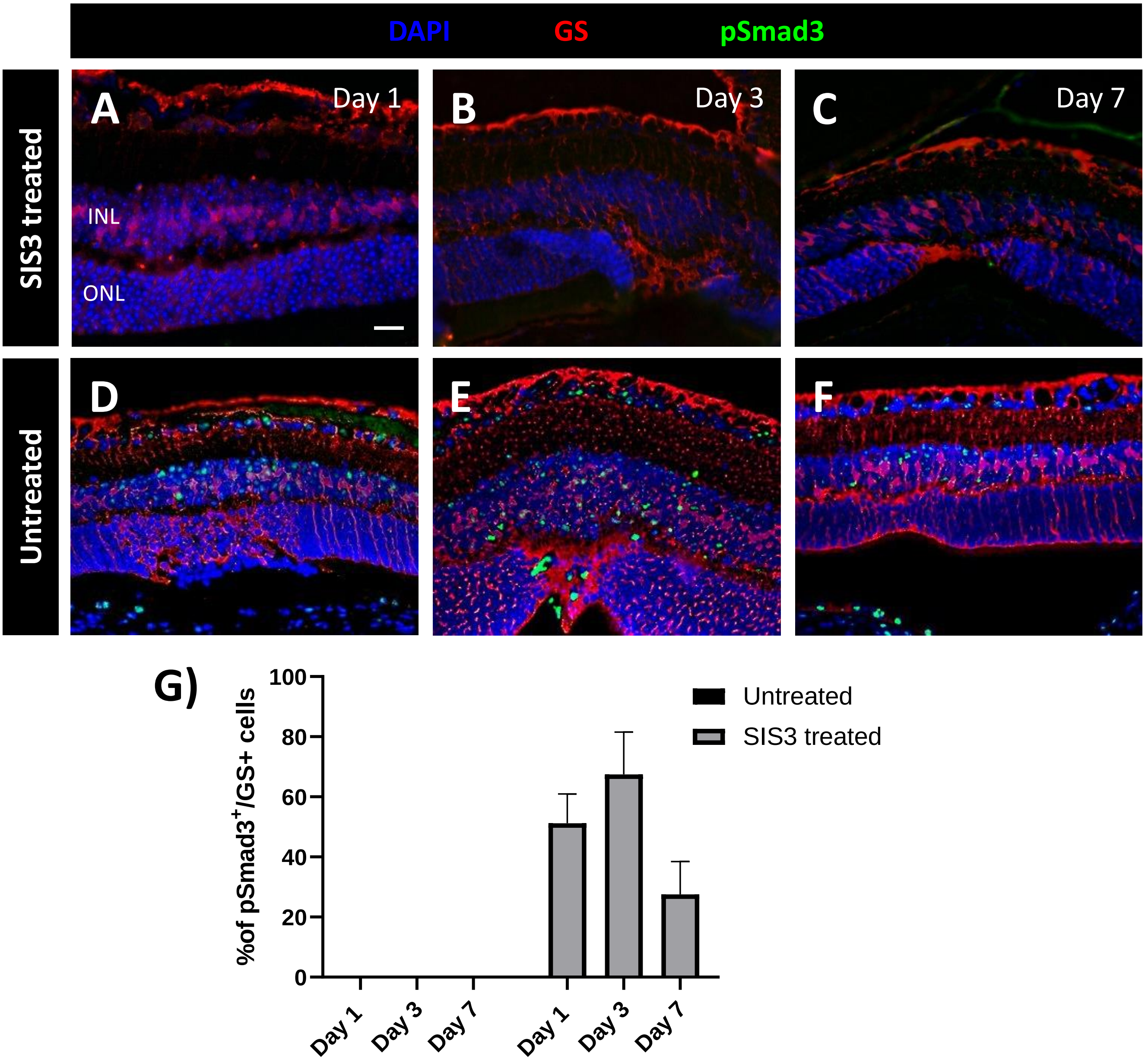

Figure S6: Efficiency of SIS3 pharmacological treatment in mice. (A-G) Analysis of pSmad3 inhibition using SIS3 in murine MC at different time points after injury (Day 1, 3 and 7). Detection of pSmad3 in GS+ MCs after laser induction in SIS3 treated (A-C) and untreated mice (D-F). Shown are representative retinal sections stained for GS (red) and pSmad3 (green). (G) Histogram illustrating the mean ± SD of the number of pSmad3+ cells normalized by the total number of GS+ cells expressed in percentage. INL, inner nuclear layer; ONL, outer nuclear layer. Scale bar of all images equals 50 μm.
